# Prolonged activation of cAMP signaling leads to endothelial barrier disruption *via* transcriptional repression of *RRAS*

**DOI:** 10.1096/fj.201700818RRR

**Published:** 2018-05-18

**Authors:** Carole Y. Perrot, Junko Sawada, Masanobu Komatsu

**Affiliations:** Cancer Center and Center for Metabolic Origins of Disease, Sanford Burnham Prebys Medical Discovery Institute at Lake Nona, Orlando, Florida, USA

**Keywords:** CREB, endothelial permeability, vascular leakage, prostaglandin E2

## Abstract

The increase in cAMP levels in endothelial cells triggers cellular signaling to alter vascular permeability. It is generally considered that cAMP signaling stabilizes the endothelial barrier function and reduces permeability. However, previous studies have only examined the permeability shortly after cAMP elevation and thus have only investigated acute responses. Because cAMP is a key regulator of gene expression, elevated cAMP may have a delayed but profound impact on the endothelial permeability by altering the expression of the genes that are vital for the vessel wall stability. The small guanosine triphosphate hydrolase Ras-related protein (R-Ras) stabilizes VE-cadherin clustering and enhances endothelial barrier function, thereby stabilizing the integrity of blood vessel wall. Here we show that cAMP controls endothelial permeability through *RRAS* gene regulation. The prolonged cAMP elevation transcriptionally repressed *RRAS* in endothelial cells *via* a cAMP response element–binding 3 (CREB3)–dependent mechanism and significantly disrupted the adherens junction. These effects resulted in a marked increase of endothelial permeability that was reversed by R-Ras transduction. Furthermore, cAMP elevation in the endothelium by prostaglandin E_2_ or phosphodiesterase type 4 inhibition caused plasma leakage from intact microvessels in mouse skin. Our study demonstrated that, contrary to the widely accepted notion, cAMP elevation in endothelial cells ultimately increases vascular permeability, and the cAMP-dependent *RRAS* repression critically contributes to this effect.—Perrot, C. Y., Sawada, J., Komatsu, M. Prolonged activation of cAMP signaling leads to endothelial barrier disruption *via* transcriptional repression of *RRAS*.

The small guanosine triphosphate hydrolase (GTPase) Ras-related protein (R-Ras) plays important roles in cellular processes, including cell adhesion to the extracellular matrix *via* integrin activation (1), endothelial cell (EC) cell–cell adhesion *via* VE-cadherin stabilization (2), and cell survival *via* PI3K/Akt pathway (3). R-Ras also attenuates ECs’ response to VEGF by inhibiting VEGF receptor 2 (VEGFR2) internalization or autophosphorylation, including the Tyr 951 phosphorylation (Tyr 949 in mouse) that is implicated in the tumor vascular permeability to permit metastatic tumor spreading (4–6). Furthermore, R-Ras elicits noncanonical Akt signaling that stabilizes microtubules in ECs and promotes endothelial lumenogenesis through this pathway (7). Significant evidence from several *in vivo* studies indicates a crucial role of R-Ras in blood vessel stabilization. R-Ras inhibits neointimal proliferation of vascular smooth muscle cells induced by arterial injury and inhibits angiogenic EC sprouting in tumors (8). R-Ras also promotes an intimate interaction between pericytes and ECs, and it stabilizes VE-cadherin clustering by limiting Ser 665 phosphorylation, which collectively enhance the integrity of blood vessel wall (2, 9). The *Rras* gene deletion in mice results in increased blood leakage in various pathologic angiogenesis models (2, 10). The up-regulation of R-Ras signaling, however, reduces vessel permeability (2). In a recent study of oxygen-induced retinopathy, R-Ras–deficient mice exhibited significantly increased blood leakage in the retina (10). This result is consistent with clinical observations of vessel leakiness in diabetic retinopathy, as R-Ras expression is found strongly suppressed in the retinal vessels of diabetic patients (10). We have previously cloned the promoter and 5′ upstream sequence of the human *RRAS* gene from ECs and partially characterized the *cis* elements that are important for the transcriptional regulation (11). However, signaling pathways to regulate the *RRAS* gene still remains largely unknown. Considering the multiple important roles that *RRAS* plays in blood vessel regeneration, maturation, and stability, it is crucial to determine the molecular mechanism underlying the regulation of this gene.

The cAMP signaling pathways are involved in a number of cellular processes, including cell growth, differentiation, and gene expression (12). Upon activation by cAMP, PKA phosphorylates transcription factors of the cAMP response element–binding (CREB)/activating transcription factor (ATF) family (13). CREB/ATF proteins binds to the cAMP response element (CRE) within promoter sequences of various genes containing the consensus TGACGTCA sequence (14). Unlike other members of the CREB family, which are activated by phosphorylation, CREB3 is anchored in the endoplasmic reticulum and is activated by intramembrane proteolysis in response to the cAMP signaling.

In ECs, cAMP regulates the endothelial permeability (15). A number of studies have demonstrated that the increase of intracellular cAMP by an adenylyl cyclase (AC) activator, forskolin, or cAMP analogs attenuates acute permeability responses of cultured ECs to platelet-activating factor or thrombin (16). It has also been shown that cAMP reduces VEGF-induced permeability in intact microvessels (17). The observed endothelial barrier stabilization by cAMP is generally attributed to the effects of cAMP on cell contractility and stability of tight/adherens junctions (18, 19). These effects are thought to be mediated in part *via* PKA-dependent phosphorylation and by inhibition of Rho GTPase and myosin light chain kinase that promotes actomyosin contraction to weaken cell–cell junctions (20). The activation of Epac, a cAMP-inducible guanine nucleotide exchange factor, enhances endothelial barrier function by stabilizing cortical actin and subsequently redistributing adherens and tight junctional molecules to cell–cell contacts *via* activation of Rap1 and its downstream Rac1 (19, 21). On the basis of these observations, it is widely accepted that cAMP signaling is barrier protective and reduces endothelial permeability. This barrier-protective effect appears to be mediated by cAMP acting at close proximity to the plasma membrane (membrane compartment) in which the adhesion molecules, effectors, and actin cytoskeleton form a junctional complex, rather than cAMP acting through CRE-dependent gene regulation. Paradoxically, several other studies have demonstrated permeability-inducing effects of cAMP (22–28). For instance, Bindewald *et al.* (25) reported that cAMP-PKA signaling can disintegrate the adhesion complexes to increase endothelial permeability and that this phenomenon is EC type dependent; the barrier disruption by cAMP was observed in microvascular ECs from coronary origin, but not in macrovascular ECs from aortic origin.

Importantly, previous studies to investigate the role of cAMP in endothelial permeability only examined the acute response to the cAMP elevation within 60 to 120 min (29, 30). The long-term effect of cAMP on EC permeability beyond this time period has not been reported. Because cAMP is a key regulator of gene expression, the elevation of intracellular cAMP may have a delayed but profound impact on the EC permeability through altering the expression of the genes that are vital for the vessel wall integrity. This important question has not been addressed previously. There are critical medical conditions such as severe sepsis or cancer that are expected to be associated with prolonged activation of cAMP signaling in the vasculature due to the high tissue levels of bacterial toxins, stress hormones, and/or prostaglandins (31–34). These conditions display considerable vascular dysfunction and vessel permeability, causing plasma leakage or cancer cell penetration into the circulation. Therefore, long-term effects of cAMP elevation have significant clinical implications.

Here we show that prolonged cAMP signaling transcriptionally represses *RRAS* *via* a CREB3-dependent mechanism and disrupts VE-cadherin at cell–cell junctions in both microvascular and macrovascular ECs. As a result, prolonged cAMP elevation ultimately leads to a marked increase in endothelial permeability.

## MATERIALS AND METHODS

### Cell culture

HUVECs, human dermal microvascular ECs (HDMECs), and EGM-2 and EGM-2-MV growth media were purchased from Lonza (Basel, Switzerland). Human brain microvascular ECs (HBECs) and Complete Human EC medium were purchased from Cell Biologics (Chicago, IL, USA). 293T cells were cultured in DMEM high glucose (Thermo Fisher Scientific, Waltham, MA, USA) supplemented with 10% fetal bovine serum and sodium pyruvate 1 mM. All our experiments were performed using HUVECs and HDMECs at passage 4 or 5.

### Plasmids

Expression vectors pCMV-N-Flag and pCMV-N-Flag-CREB1 encoding a full-length cDNA for human CREB1 flanked with an N-terminal Flag tag were purchased from Sino Biological (Beijing, China). P3X-Flag-CREB3/LZIP encoding a full-length cDNA of human CREB3/LZIP with N-terminal Flag tags was obtained from Addgene (Cambridge, MA, USA). The human *RRAS* promoter–luciferase reporter construct in pGL3 vector pGL3-*RRAS*-1907/+1-Fluc was described by Xu and Komatsu (11). Mutations in the promoter sequence were carried out using GenScript (Piscataway, NJ, USA) by deletion of CRE. Lentiviral reporter vector pLenti6-R4R2-*RRAS*-1907/+1-Fluc was generated by subcloning the *RRAS* promoter–reporter construct into the pLenti6 vector in order to stably transduce ECs.

### Antibodies and reagents

For Western blot analysis, rabbit anti–R-Ras antibody was obtained from AnaSpec (Fremont, CA, USA). Rabbit anti-CREB1, anti–activating transcription factor 2 (ATF2), and anti–PKACα were purchased from Cell Signaling Technology (Danvers, MA, USA). Rabbit anti–VE-cadherin and mouse anti-GAPDH were purchased from Santa Cruz Biotechnology (Dallas, TX, USA). All secondary horseradish peroxidase–conjugated antibodies were purchased from Promega (Madison, WI, USA). Mouse anti-Flag antibody used for chromatin immunoprecipitation (ChIP) was obtained from MilliporeSigma (Burlington, MA, USA). For immunofluorescence staining, rabbit anti-CD31, mouse anti-cAMP, and rabbit anti-fibrinogen were obtained from Abcam (Cambridge, MA, USA), rat anti-mouse CD31 and mouse anti-human CD31 from BD Biosciences (San Jose, CA, USA), mouse anti-R-Ras clone 2E12 from Abnova (Taipei, Taiwan), rabbit anti-phospho-CREB1^Ser133^ from Cell Signaling Technology, and mouse anti–VE-cadherin Santa Cruz Biotechnology. Alexa Fluor secondary antibodies (488, 555, and 647) were from Thermo Fisher Scientific. Mouse VE-cadherin antibody (clone BV6) used for VE-cadherin internalization assay was purchased from MilliporeSigma. Forskolin was obtained from Calbiochem (San Diego, CA, USA), 8-bromo-cAMP from Abcam, and 3-isobutyl-1-methylxanthine (IBMX) and prostaglandin E_2_ (PGE2) from Tocris Bioscience (Bristol, United Kingdom).

### Western blot analysis

Western blot analysis was performed by electrophoresis of cell lysate on Mini-Protean TGX Precast Gels (Bio-Rad, Hercules, CA, USA), followed by electrotransfer to nitrocellulose membrane (Bio-Rad). After blocking unspecific binding, antibody incubations were carried out overnight in blocking buffer (5% bovine serum albumin in Tris-buffered saline containing 0.1% Tween 20), and target proteins were detected using Western Lightning Plus-ECL (PerkinElmer, Waltham, MA, USA).

### RNA extraction and real-time quantitative RT-PCR

RNA extraction was performed using the Nucleospin RNA kit (Macherey-Nagel, Düren, Germany). Between 500 ng and 1 µg of RNA was then subjected to DNase I digestion (Thermo Fisher Scientific) followed by reverse transcription using the iScript cDNA Synthesis Kit (Bio-Rad). Quantitative PCR (qPCR) was then performed using Power SYBR Green PCR Master Mix (Thermo Fisher Scientific). Primer sets for R-Ras, CREB1, ATF2, CREB3, PGE2 receptor 4 (EP_4_), and cyclophilin A are shown in **Table 1**.

**TABLE 1:** Primer sets used for qPCR and ChIP-qPCR experiments

Primer	Sequence, 5′–3′	
	Forward	Reverse
Primer sets for qPCR		
* RRAS*	TAACGACCGGCAGAGTTTCA	ACCAACACAACGGGGAAGTC
* CREB3*	CTTTCTGAGGTACCGAGCGACTG	GAGAATGTTCAACGACGCTGG
*EP4*	AGGACAAGGTGAAAGCAGGTT	AGTGCAAGGCTGGGTCTGTAG
* Cyclophilin A*	CAAATGCTGGACCCAACACA	TGCCATCCAACCACTCAGTCT
Primer sets for ChIP-qPCR		
−1887F	CAGCTAATGGGGTGAGAGGT	
−1741R		GACTACAATTCCCTTGCGTC
−965F	AGCCTCTCCAATTCCTGGAC	
−885R		GTAGACAGGTTCCTTCGTAC
−402F	CCAAGCAGCAGTGTCACAG	
−292R		TCTGTGGGCGTGGCTATG

### Cell proliferation assay

Cell proliferation was assessed by the (4,5-dimethylthiazol-2-*yl*)-2,5-diphenyltetrazolium bromide (MTT) Cell Proliferation Assay Kit (Cayman Chemicals, Ann Arbor, MI, USA). HUVECs were seeded in 96-well plates (5000 cells/well) and treated 24 h later with forskolin, IBMX, or 8-bromo-cAMP for an additional 48 h. Cells were then incubated with MTT reagent (10 µl in 100 µl cell culture medium) for 4 h at 37°C, followed by 18 h incubation with 100 µl of crystal dissolving solution at 37°C. Absorbance was measured in 4 different wells at 570 nm. The experiment was repeated twice.

### Cell viability assay

Cell viability was analyzed using the CellTiter Fluor Cell Viability Assay Kit (Promega). HUVECs were cultured and treated in the same conditions as for MTT assay; they were incubated with 20 µl of substrate in 80 µl cell culture medium for 1 h at 37°C. Results were obtained by measuring fluorescence signals from 4 different wells. The experiment was repeated twice.

### ELISA

To determine intracellular cAMP levels, HUVECs were seeded in 12-well plates at a density of 60,000 cells/well for 24 h, then treated with forskolin or IBMX for various periods of time. Cell lysates were collected by adding 0.5 ml of HCl 0.1 N in each well, and samples were tested using the Direct cAMP ELISA Kit for Intracellular Quantification according to the manufacturer’s instructions (Enzo Life Sciences , Farmingdale, NY, USA).

For the quantification of VEGF-A^165^ in the conditioned media, HUVECs were cultured as previously described and subjected to 48 h forskolin treatment. The conditioned medium was then collected from each well, and VEGF-A^165^ concentration was determined by the Human VEGF ELISA Kit according to the manufacturer’s instructions (Thermo Fisher Scientific). All conditions were tested in at least 3 independent wells, and experiments were repeated at least twice.

### Chromatin immunoprecipitation

293T cells transfected with either pCMV-N-Flag-CREB1 or p3X-Flag-CREB3/LZIP were grown to 90% confluence in 15 cm diameter plates and fixed with a 1% formaldehyde solution. Then ChIP was carried out according to a detailed protocol. In brief, 25 µg of sonicated sheared chromatin were incubated overnight at 4°C with 5 µg of mouse anti-Flag antibody, then incubated for 2 h with protein G–agarose/salmon sperm DNA beads (MilliporeSigma). Precipitated DNA was purified by phenol/chloroform extraction and processed for qPCR analysis of the regions of interest within the *RRAS* promoter sequence using Power SYBR Green PCR Master Mix (Thermo Fisher Scientific). ChIP-qPCR results were calculated using the ∆∆*C_t_* method and are shown as percentages of input DNA for each ChIP experiment. PCR primer sequences are listed in Table 1.

### Lentivirus transduction

For reporter assays, HUVECs and HDMECs were stably transduced with pLenti6-R4R2-*RRAS*-1907/+1-Fluc lentivirus (multiplicity of infection 0.5; hexadimethrine bromide 10 µg/ml). For R-Ras rescue experiments, cells were transduced with a lentiviral expression vector carrying cDNA for a constitutively active form of R-Ras (R-Ras38V), wild-type (WT) R-Ras, or insertless control (mock) as described by Komatsu and Ruoslahti (8). The day after transduction, media were replaced with fresh media, and the cells were cultured for 3 d before their use in experiments.

### RNA interference

For gene silencing, cells were transfected at ∼60% confluence (60,000 cells/well in 12-well plates) in complete medium with 30 nM small interfering RNA (siRNA) targeting CREB1 (Cell Signaling Technology), ATF2 (Cell Signaling Technology), CREB3 (Thermo Fisher Scientific), or catalytic α subunit of PKA (PKACα; Cell Signaling Technology) using Lipofectamine RNAiMax Reagent (Thermo Fisher Scientific). For gene silencing followed by reporter assays, cells were first transduced with the *RRAS*-1907/+1-Fluc lentivirus and then transfected with siRNA in 96-well plates at a density of 15,000 cells/well. Luciferase activity was measured 72 h later using the Dual-Glo Luciferase Kit from Promega.

### Transient transfection

293T cells were transfected at ∼60% confluence (5000 cells/well in 96-well plates) in complete medium with Lipofectamine 2000 (Thermo Fisher Scientific). pRL-TK reporter vector (*Renilla* luciferase; Promega) was used as an internal control to monitor transfection efficiency. Luciferase activity was assessed 48 h after transfection using the Dual-Glo luciferase assay system (Promega) and a PerkinElmer plate reader.

### Immunofluorescence

EC monolayers were fixed with 4% paraformaldehyde for 10 min, then permeabilized using PBS solution containing 5% fetal bovine serum and 0.1% Triton X-100 for 1 h at room temperature. Cells were incubated with mouse anti–VE-cadherin antibody (1/200 dilution) overnight at 4°C, followed by incubation with anti-mouse Alexa Fluor 488 secondary antibody (1/1000) for 2 h at room temperature. Slides were then mounted with coverslips, and signals were visualized using a fluorescence microscope (Eclipse 90i; Nikon, Tokyo, Japan). Images were captured using NIS-Elements BR3.2 software (Nikon) and analyzed by ImageJ software (Image Processing and Analysis in Java; National Institutes of Health, Bethesda, MD, USA; *https://imagej.nih.gov/ij/*). Quantification of fluorescence was performed by measuring the integrated intensity of 3 different areas, divided by the number of cells present in these areas.

### Analysis of VE-cadherin internalization

The VE-cadherin internalization assay was performed as previously described (2, 9), with a minor modification. Briefly, cells were seeded onto Nunc Lab-Tek chamber slide (20,000 cells/well) for 24 h. Cells were next treated with forskolin (50 µM) or IBMX (1 mM) for 48 h, then incubated with 1 μg/ml monoclonal anti-human VE-cadherin antibody (clone BV6) for 1 h with gentle shaking. This incubation was carried out at 4°C to block intracellular trafficking. Cells were then incubated at 37°C for 30 min to allow internalization of cell surface VE-cadherin. Subsequently, the cells were washed with mild acid buffer (25 mM glycine in PBS, pH 2.8) for 15 min at 4°C with agitation in order to remove bound antibodies from the cell surface. After fixation and membrane permeabilization, cells were stained with anti-mouse Alexa Fluor 488 (1/1000) and Alexa Fluor 594–conjugated phalloidin (1/1000) to visualize internalized VE-cadherin.

### Endothelial permeability assay

To study short-term effect of cAMP pathway activation on endothelial permeability, HUVECs were plated at 2 × 10^4^ cells/well into the 24-well Transwell chamber (0.4 μm pore size; Corning, Corning, NY, USA), then cultured in EGM-2 (Lonza) to produce an EC monolayer. After 24 h, cell culture medium was replaced in both the lower and upper chambers with phenol red–free EC growth basal medium 2 (EBM-2) containing forskolin (50 µM), IBMX (1 mM), or control DMSO. FITC-labeled 70 kDa dextran (MilliporeSigma) was immediately added into the upper chamber at a final concentration of 1 mg/ml. At various time points, the fluorescence intensity in the lower compartment was measured by a fluorophotometer. The percentage permeability of the endothelial monolayer was determined with the fluorescent intensity of the empty chamber without cells as 100% permeability. To analyze the long-term effect of cAMP pathway activation, HUVECs or HDMECs were plated at 2 × 10^4^ cells/well into the 24-well Transwell chamber (0.4 μm pore size; Corning) and grown to confluence for 24 h in EGM-2 growth medium (Lonza). Cells were treated with forskolin (50 µM), IBMX (1 mM), or control DMSO for additional 48 h, and culture medium was replaced in both lower and upper chambers with phenol red–free EBM-2. FITC-labeled 70 kDa dextran was added at 1 mg/ml final concentration to the upper chamber. At various time points, the fluorescence intensity in the lower compartment was measured by a fluorophotometer. The percentage permeability of the endothelial monolayer was determined with the fluorescent intensity of the empty chamber without cells as 100% permeability. To investigate the role of CREB3 in the long-term effect of cAMP pathway on endothelial permeability, HUVECs were cultured in 24-well Transwell chambers for 24 h to produce a confluent EC monolayer and subsequently transfected overnight with control or CREB3 siRNA. The culture medium was replaced with fresh EGM-2, and cells were treated with DMSO or forskolin (50 µM) for 48 h. After replacing the cell culture medium with phenol red–free EBM-2, FITC-dextran (70 kDa, 1 mg/ml) was added to the upper chamber, and the fluorescence signal in the lower compartment was measured at different time points. Experiments were conducted with duplicate wells for each condition and were repeated 3 times to confirm reproducibility.

### *In vivo* permeability assay

Eight-week-old C56BL/6 mice were injected intradermally with vehicle (30% DMSO in PBS), IBMX (300 µg/kg), or PGE2 (20 or 200 ng) on d 0 and d 1 in the same area of dorsal skin. On d 2 (48 h from the first IBMX or PGE2 injection), mice received an intravenous injection of 2000 kDa FITC-dextran (1 mg/ml) from the tail vein. The animals were humanely killed 30 min later. Skin samples were collected and cryopreserved in optimal cutting temperature compound for preparation of tissue sections. Tissue sections were fixed with 4% paraformaldehyde for 15 min at room temperature, followed by incubation in blocking buffer (10% goat serum, 3% bovine serum albumin in PBS) for 1 h at room temperature, then immunostained for CD31 (1/100) or for CD31 and cAMP (1/200) overnight at 4°C. Next, tissue sections were rinsed and incubated with anti-rabbit Alexa Fluor 555 and anti-mouse Alexa Fluor 647 secondary antibodies (1/500) for 2 h at room temperature. Fluorescence images were captured by NIS-Elements BR3.2 software and analyzed by ImageJ and Volocity software. The extent of plasma leakage was determined by integrated fluorescence intensity of FITC-dextran leaked outside the CD31-stained vessels. For this, the fluorescence intensity of the FITC-only positive area (extravasated FITC-dextran) was standardized for the vessel count in the area analyzed. Apoptotic cells in the skin tissue sections were identified with the SignalStain Apoptosis (Cleaved Caspase-3) IHC Detection kit (Cell Signaling Technology) to detect activated caspase-3 using a cleaved caspase-3 (Asp175) (D3E9) rabbit mAb. Images were captured by Aperio ImageScope software (Leica, Wetzlar, Germany). The plasma leakage and cell apoptosis analyses were conducted for 15 ∼ 21 (for IBMX) or >40 (for PGE2) micrographs of different areas in 4 skin samples. The skin tissue sections were also examined histologically for accumulation of infiltrating immune cells.

### RNA sequencing analysis

Human lung microvascular ECs were obtained from Lonza and were cultured in EGM-2-MV. To remove lymphatic ECs, cells were stained with anti-podoplanin antibody (eBioscience, San Diego, CA, USA) and then were fluorescence-activated cell sorted (BD FACS Aria II Sorter; BD Biosciences) to collect podoplanin-negative cells. The sorted cells were cultured for an additional 3 d, and the total RNA was extracted for RNA sequencing. To construct multiplexed Libraries, 1 µg of total RNA was used for TruSeq Stranded Total RNA Library Prep Kit (Illumina, San Diego, CA, USA). Multiplexed libraries were pooled, and sequencing was performed on one flow cell of an Illumina HiSeq 2500. The FastQ file from sequencing was uploaded to Partek Flow (Partek, Chesterfield, MO, USA). The sequence read was mapped to whole-genome hg19 using TopHat 2 2.1.0 (*https://ccb.jhu.edu/software/tophat/index.shtml*) and quantified on the basis of a RefSeq transcript model (August 4, 2015) (**Table 2**).

**TABLE 2:** RNA sequence analysis of human lung microvascular ECs

Gene	Reads/kilobase/10^6^	Gene	Reads/kilobase/10^6^
*CREB1*	0.651	*TIE1*	13.806
*ATF2*	0.536	*TEK*	0.992
*CREB3*	7.279	*ANGPT1*	0.003
*ADRA1A*	0.001	*ANGPT2*	1.136
*ADRA1B*	0.034	*ANGPT4*	0.001
*ADRA1D*	0.004	*RAC1*	6.656
*ADRA2A*	0.058	*RAC2*	3.179
*ADRA2B*	0.017	*RAC3*	3.256
*ADRA2C*	0.000	*RHOA*	8.227
*ADRB1*	0.029	*RHOB*	111.569
*ADRB2*	2.764	*RHOC*	61.072
*ADRB3*	0.015	*RHOD*	0.302
*ICAM1*	2.034	*RHOF*	0.213
*CDH5*	19.472	*RHOG*	1.435
*PECAM1*	64.257	*RHOH*	0.001
*FLT1*	0.883	*RAP1A*	0.217
*KDR*	9.786	*RAP1B*	2.889
*FLT4*	0.181	*S1PR1*	32.396
*VEGFA*	0.434	*S1PR2*	0.103
*VEGFB*	3.146	*S1PR3*	1.785
*VEGFC*	0.187	*S1PR4*	0.228
*RRAS*	5.414	*S1PR5*	0.011
*TC21*	0.575	*TGFBI*	2.355
*MRAS*	0.088	*TGFB2*	0.142
*NRAS*	13.365	*TGFB3*	0.081
*KRAS*	0.858	*TGFBR1*	0.398
*HRAS*	8.150	*TGFBR2*	5.635
*AKT1*	5.101	*TGFBR3*	0.096
*AKT2*	0.325	*SMAD1*	0.608
*AKT3*	0.255	*SMAD1-AS1*	0.086
*JAG1*	2.811	*SMAD1-AS2*	0.047
*JAG2*	1.295	*SMAD2*	1.148
*JAGN1*	5.149	*SMAD3*	0.637
*DLL1*	0.1397	*SMAD4*	1.070
*DLL3*	0.0467	*SMAD5*	1.648
*DLL4*	1.7064	*SMAD5-AS1*	0.104
*NOTCH1*	1.543	*SMAD6*	0.042
*NOTCH2*	0.361	*SMAD7*	0.086
*NOTCH2NL*	0.137	*SMAD9*	0.100
*NOTCH3*	0.006	*ACVRL1*	15.708
*NOTCH4*	7.280	*TGFBR1*	0.398

RNA-Seq analysis was conducted to compare relative expression levels of angiogenesis or vascular stability-related genes and other genes in ECs.

### Breast tissue specimens and immunostaining

Tissue samples from breast cancer patients who underwent mastectomy for adenocarcinoma were provided by the Florida Hospital Cancer Institute. Frozen tissue blocks embedded in Optimal Cutting Temperature compound were sectioned to 12 µm thickness and immunostained for CD31 (1/200) and phospho-CREB1 (1/200), R-Ras (1/100), or fibrin (1/200) overnight at 4°C. Next, tissue sections were rinsed and incubated with anti-mouse Alexa Fluor 488 or 555 and anti-rabbit Alexa Fluor 488 or 555 secondary antibodies (1/500) for 2 h at room temperature. Fluorescence images were captured by NIS-Elements BR3.2 software and analyzed by ImageJ software. The sections of breast cancer tissues and noncancerous normal portion of breast tissues from the same patients were examined. Samples from 4 breast cancer patients were analyzed.

### *In silico* promoter analysis

The software tool MatInspector (Genomatix, Munich, Germany) was used to identify putative transcription factor binding sites within the *RRAS* promoter sequence (3.2 kb upstream relative to the transcription start site). All analyses were conducted with threshold values core similarity 1.0 and matrix similarity >0.75.

### Statistical analysis

Statistical analysis was performed by GraphPad Prism 7 (GraphPad Software, La Jolla, CA, USA). The 2-tailed Student’s *t* test was used to compare 2 conditions. For multiple comparisons, we used 1-, 2-, or 3-way ANOVA with Dunnett’s or Tukey’s multiple comparison test as appropriate for each comparison. Error bars represent sem.

## RESULTS

### Elevation of intracellular cAMP transcriptionally suppresses R-Ras expression in ECs

**Figure 1:**
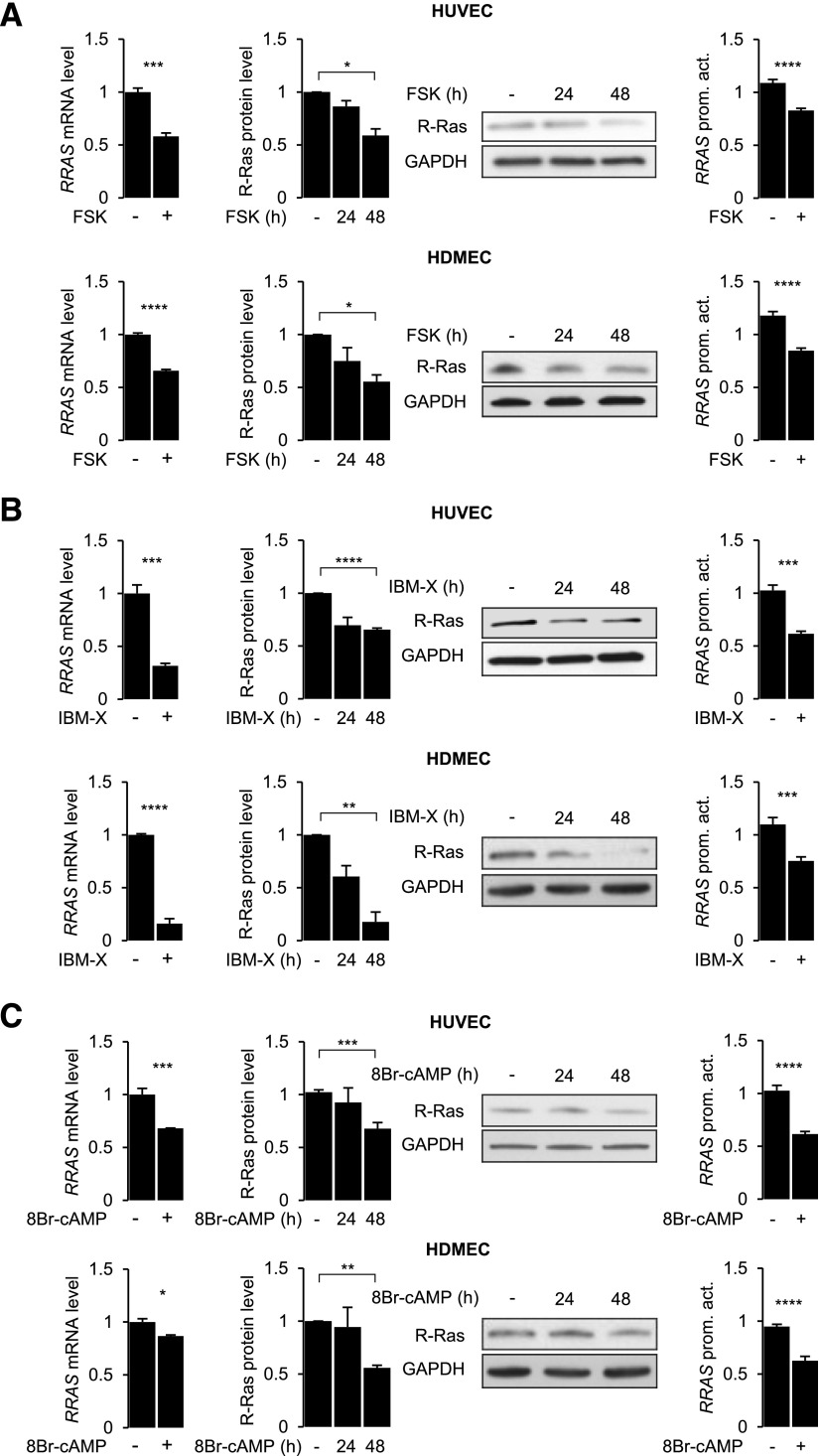
Prolonged activation of cAMP pathway down-regulates R-Ras expression in ECs. HUVECs and HDMECs were treated with forskolin (*A*; 50 µM), IBMX (*B*; 1 mM), or 8-bromo-cAMP (*C*; 250 µM), and R-Ras expression was analyzed by qRT-PCR at 24 h and by Western blot at 24 and 48 h after treatment. In parallel, luciferase assay was conducted to determine activity of −1907/+1 *RRAS* promoter–reporter construct after 24 h treatment of HUVECs with same compounds. qRT-PCR was repeated in at least 3 independent experiments and in triplicate. Luciferase assay results are shown as means ± sem of 3 independent experiments performed in triplicate or quadruplicate. ****P < 0.0001, ***P < 0.001, **P < 0.01, *P < 0.05 (1-way ANOVA).

We investigated the effect of cAMP signaling on R-Ras expression in cultured HUVECs and human dermal microvascular ECs (HDMECs) (**Fig. 1**). To activate cAMP signaling, we treated these ECs with chemicals known to elevate the cellular level of cAMP {*i.e.*, forskolin (AC activator) (Fig. 1*A*), IBMX [phosphodiesterase (PDE) inhibitor] (Fig. 1*B*), or 8-bromo-cAMP (PDE-resistant cAMP analog) (Fig. 1*C*)}. Real-time qPCR and Western blot analyses were conducted to determine the R-Ras mRNA and protein levels at 24 and 48 h after cAMP signaling activation. These analyses demonstrated that R-Ras mRNA and protein levels were down-regulated by the cAMP signaling in both macrovascular (HUV) and microvascular ECs (Fig. 1).

We next examined the effect of forskolin, IBMX, or 8-bromo-cAMP on the *RRAS* promoter activity to determine whether the regulation of R-Ras expression by cAMP is mediated at the transcriptional level. We previously isolated human *RRAS* promoter/upstream sequence from ECs and constructed *RRAS* promoter–luciferase reporter plasmids with various lengths of the *RRAS* promoter sequence (11). One of these constructs (clone Hsp6; −1907/+1 *RRAS*-luc) was subcloned into a lentivirus expression vector for transduction into cultured ECs. The HUVECs and HDMECs transduced with this promoter–reporter construct were treated with forskolin, IBMX, or 8-bromo-cAMP for 24 h (Fig. 1). In this study, we found that the *RRAS* promoter activity was suppressed by all 3 cAMP-elevating compounds in both EC cell types. A PDE-4–specific inhibitor, rolipram, also inhibited *RRAS* promoter activity in HUVECs (data not shown). We observed that the treatment of ECs with forskolin, IBMX, or 8-bromo-cAMP for 48 h did not affect cell proliferation or viability, except for a modest decrease of proliferation by 8-bromo-cAMP (Supplemental Fig. S1*A, B*). ELISA analyses demonstrated a prolonged elevation of intracellular cAMP that lasts more than 48 h in HUVECs treated with forskolin or IBMX (Supplemental Fig. S1*C*).

### cAMP regulates R-Ras expression through CREB family member CREB3

**Figure 2:**
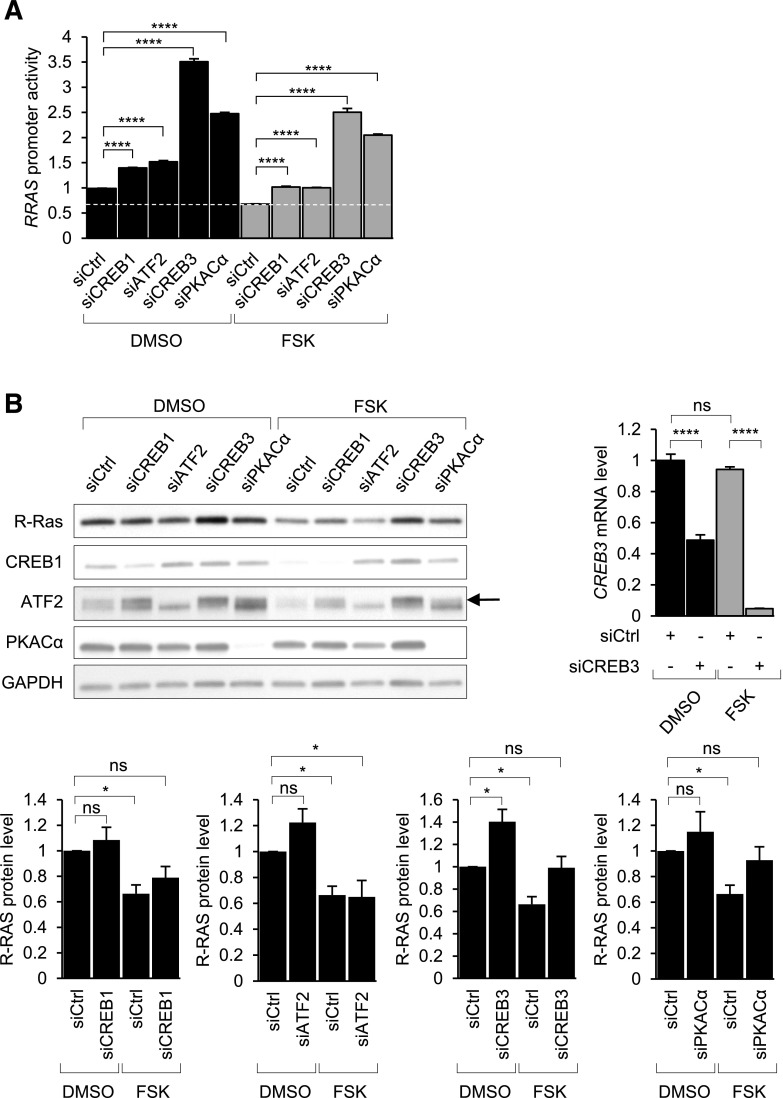
cAMP represses *RRAS* gene expression mainly *via* CREB3. *A*) Luciferase reporter assays were performed to determine *RRAS* promoter activity in HUVECs in which cAMP effectors were silenced by siRNA for 48 h. Results are shown as means ± sem of 3 independent experiments performed in triplicate. ****P < 0.0001 (2-way ANOVA). *B*) Protein levels of R-Ras, PKA catalytic-α, CREB1, and ATF2 were determined by Western blot analysis. Knockdown of CREB3 was assessed by qRT-PCR due to lack of availability of good antibody for this purpose. Effect of cAMP effector silencing on R-Ras protein level was quantified by ImageJ software (bottom graphs). Arrow in upper band indicates ATF2. Ns, not significant. *P < 0.05, ****P < 0.0001 (2-way ANOVA).

PKA and the CREB/ATF family members mediate cAMP-dependent gene regulations. We investigated the role of PKA and CREB/ATF proteins in the transcriptional regulation of R-Ras by knocking down these proteins using siRNA. The knockdown of CREB1 or ATF2 individually resulted in a moderate increase (≤50%) of the *RRAS* promoter activity in the presence or absence of forskolin for 24 h (**Fig. 2*A***). In contrast, the knockdown of CREB3 or PKACα markedly increased the *RRAS* promoter activity in the same conditions. Moreover, CREB3 knockdown restored the R-Ras protein level in the forskolin-treated cells (Fig. 2*B*). PKACα knockdown had a similar effect on the R-Ras protein level. In contrast, the knockdown of other CREB proteins had little effect on the protein level (Fig. 2*B*). CREB1 and ATF2 appears to play a minor role in the *RRAS* promoter regulation. The CREB3 knockdown increased CREB1 and ATF2 expression, presumably to compensate for the reduction of CREB3 (Fig. 2*B*). However, these increases were insufficient to restore the suppressive effect of cAMP. These results demonstrate that CREB3 is the most important among these CREB proteins for *RRAS* gene regulation. In an RNA sequencing analysis, we found that CREB3 is expressed >10-fold more abundantly than CREB1 and ATF2 in human microvascular ECs, further supporting the importance of CREB3 in EC regulations (Table 2).

### CREB proteins bind to 2 distinct CRE-containing regions within *RRAS* promoter

Because R-Ras is transcriptionally repressed by cAMP signaling predominantly through CREB3, we searched for putative CREB binding sites in a ∼9 kb region (position −3200 to 5761 from putative transcription start site) of the human *RRAS* gene sequence including exons, introns, and up- or downstream distal regions. An *in silico* analysis by Genomatix software identified the presence of 3 putative CREs located at positions −1843, −1791 (on the positive strand), and −348 (on the negative strand). The 2 most distal sites contain the CRE core sequence 5′-TGACG-3′, while the proximal site harbors an incomplete 5′-GACG-3′ core sequence (**Table 3**). The Genomatix program predicted 2 additional potential CRE sites for binding of c-Jun/ATF2 and ATF1 within the second intron and downstream of the last (sixth) exon, respectively. However, because the analyses of CREB family protein silencing suggested that CREB3 is the main mediator of R-Ras down-regulation by cAMP signaling (Fig. 2*B*), we focused on the 3 upstream CREB binding sites (−1843, −1791, and −348) for subsequent studies.

**TABLE 3:** Putative CREs within human RRAS promoter identified in silico

Strand	Start position	End position	Core similarity score	Matrix similarity score	CRE sequence, 5′–3′
+	−1843	−1823	1	0.907	CCGCGATGACGCCCCACAGTG
+	−1791	−1771	1	0.989	AGGAGGTGACGTAAGAGGCGC
−	−348	−328	1	0.756	TTCAGCAGACGGGGCGGAAAG

CREs were predicted using MatInspector from Genomatix. Position of CREs is relative to methionine start codon of *Rras* gene. Strands (+) and (−) are relative to same or opposite *RRAS* gene-carrying DNA strand, respectively. “Core sequence” of matrix is defined as (usually 4) consecutive highest conserved positions of matrix. Maximum core similarity of 1.0 is only reached when highest conserved bases of a matrix match exactly in sequence. Matrix similarity takes into account all bases over whole matrix length. Perfect match to matrix gets a score of 1.00 (each sequence position corresponds to highest conserved nucleotide at that position in matrix); good match to matrix usually has a similarity of >0.80. Source: https://www.genomatix.de/online_help/help/scores.html. *In silico* analysis identified 3 putative CREs (core sequence 5′-TGACG) within −1907/+1 region of human *RRAS* promoter. Core sequences are underlined.

**Figure 3:**
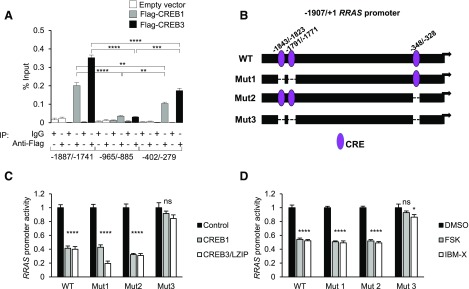
*RRAS* promoter contains 3 CREs that are necessary for CREB-driven transcriptional repression. *A*) CREB1 and CREB3 binding to different regions containing CRE sequences was assessed by ChIP-qPCR in 293T cells transfected with empty or Flag-tagged human CREB1 or CREB3. DNA enrichment in each immunoprecipitation sample is represented as percentage of total input chromatin. **P < 0.01, ***P < 0.001, ****P < 0.0001 (1-way ANOVA). *B*) Schematic representations of WT and mutant constructs of −1907/+1 *RRAS* promoter showing locations of 3 CREs. Promoter mutants were generated by deletion of 2 distal CREs (Mut1), most proximal CRE (Mut2), or all 3 CREs (Mut3). *C*) 293T cells were cotransfected with one of the *RRAS* promoter–reporter constructs together with CREB1 or CREB3 expression plasmid. Luciferase activity was measured 48 h later. *D*) 293T cells were transfected with various *RRAS* promoter constructs and subsequently treated with DMSO (control), forskolin, or IBMX for 24 h before measuring luciferase activity. Ns, not significant. ****P < 0.0001, *P < 0.05 (1-way ANOVA).

To assess the binding of CREB proteins to these sites, we performed ChIP after the cDNA transfection of Flag-tagged CREB1 or CREB3 into 293T cells. qPCR amplification after anti-Flag immunoprecipitation revealed that the CREB proteins bind to both −1887/−1741 and −402/−279 regions containing the CREs identified *in silico* (**Fig. 3*A***). CREB3 showed substantially more efficient binding to the −1887/−1741 region than CREB1. The CREB1 and CREB3 binding to the −965/−885 sequence that lacks CREs was negligible, demonstrating the site specificity of CREB binding. Interestingly, the binding of CREB3 to the −1887/−1741 region was 2-fold greater than the binding to the −402/−279 region. This is likely due to the presence of 2 CREs in the −1887/−1741 region. In contrast, CREB1 bound to the two regions at a similar level. These results suggest that either one or both of the two CREs in the −1887/−1741 region may have a much lower affinity for CREB1 than for CREB3, further supporting the significance of CREB3 for the *RRAS* gene regulation.

To gain functional insights into the regulatory role of these CREs, we inactivated these sites by deletion. The CREs located at positions −1843 and −1791 were deleted altogether from the *RRAS* promoter (*RRAS^Mut1^*). The other CRE at position −348 was deleted independently (*RRAS^Mut2^*). A third mutant promoter construct with deletions of all 3 CREs was also generated (*RRAS^Mut3^*) (Fig. 3*B*). We studied the activity of these mutant and WT *RRAS* promoters in 293T cells that were cotransfected with control, CREB1, or CREB3 expression vectors. In this analysis, CREB1 and CREB3 repressed the *RRAS^WT^*, *RRAS^Mut1^*, and *RRAS^Mut2^* promoter activities (Fig. 3*C*). However, neither CREB1 nor CREB3 was able to suppress the *RRAS^Mut3^* promoter activity (Fig. 3*C*). Next, we examined the activities of the WT and mutant *RRAS* promoters after forskolin or IBMX treatment of the cells. Individual deletion of the distal (Mut1) or proximal (Mut2) region did not affect the *RRAS* promoter repression by forskolin or IBMX (Fig. 3*D*). However, the deletion of all 3 CREs abolished the forskolin- or IBMX-induced suppression of *RRAS* promoter activity (Fig. 3*D*). Taken together, these results demonstrated that both distal and proximal CREs are the regulatory elements responsible for the transcriptional suppression of the *RRAS* gene in response to cAMP signaling.

### Prolonged cAMP signaling disrupts VE-cadherin and endothelial barrier

One of the important functions of R-Ras is to enhance the blood vessel wall integrity and reduce blood leakage by stabilizing VE-cadherin at the endothelial adherens junction (2, 10). In the current study, we showed compelling evidence that cAMP is a negative regulator of R-Ras expression. It is therefore paradoxical that there have been many reports of endothelial barrier stabilization by cAMP elevation. Further confusing the precise role of cAMP, other reports have described the barrier disruption of by cAMP in microvascular ECs but stabilization in macrovascular (aortic) ECs (25, 35). Notably, all of these observations were made within less than a few hours of cAMP elevation and before cAMP signaling could affect cell behavior through gene regulation.

**Figure 4:**
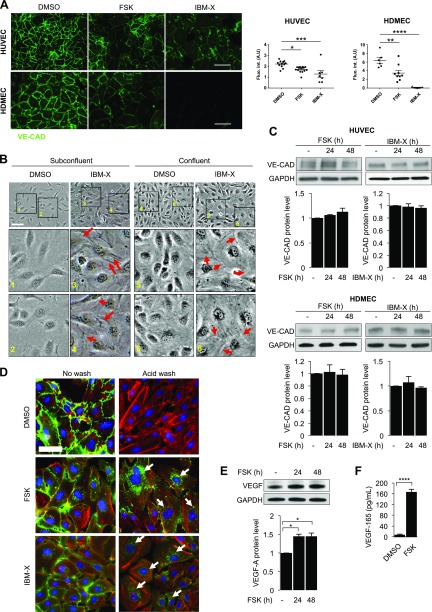
Prolonged cAMP signaling results in disruption of VE-cadherin and breakdown of endothelial barrier. *A*) Confluent monolayer cultures of HUVECs (top) and HDMECs (bottom) were treated with DMSO, forskolin, or IBMX for 48 h then stained for VE-cadherin (VE-CAD). Quantification of immunostaining was performed by measuring integrated fluorescence intensity of 3 distinct areas from at least 4 micrographs for each condition by ImageJ software. Scale bars, 100 µm. *P < 0.05, **P < 0.01, ***P < 0.001, ****P < 0.0001 (1-way ANOVA). *B*) Phase-contrast micrographs of subconfluent and confluent HUVEC monolayers treated with or without IBMX for 48 h. Bottom image provides magnification of indicated areas. Arrows indicate special gap formation between adjacent ECs exhibiting detachment of cells from each other. In contrast, cell–cell boundaries are unrecognizable in control culture. Scale bar, 50 µm. *C*) Effect of forskolin (FSK) or IBMX on VE-cadherin expression was assessed by Western blot analysis at 24 and 48 h. Top: HUVECs; bottom: HDMECs. *D*) Analysis of VE-cadherin internalization. Monoclonal antibody (BV6) recognizes extracellular domain of VE-cadherin (green). After antibody incubation in EC culture, cell-surface membrane-bound BV6 antibodies (left) were removed by mild acid wash (right). Presence of internal acid-resistant vesicles stained with BV6 antibody indicates internalization of VE-cadherin (arrows). Phalloidin (actin), red; nucleus, blue. Scale bar, 50 µm. *E*) Western blot analysis of VEGF-A expression was performed in HUVECs treated with forskolin for 24 or 48 h. Band intensity was quantified by ImageJ software. *P < 0.05 (1-way ANOVA). *F*) Concentration of VEGF-A^165^ in conditioned media of HUVEC cultures treated with DMSO or forskolin for 48 h was quantified by ELISA. ****P < 0.0001 (Student’s *t* test).

To understand the precise role of cAMP in endothelial integrity, we investigated the long-term effect of cAMP elevation on macro- and microvascular ECs. HUVECs and HDMECs were treated with forskolin or IBMX for 48 h, and the cell–cell junctional stability was analyzed by immunofluorescence staining of VE-cadherin. This study demonstrated that cAMP elevation significantly diminished VE-cadherin fluorescence signal at the cell–cell boundaries, indicating reduced VE-cadherin clustering at adherens junctions (**Fig. 4*A***). In the control culture, neighboring ECs formed intimate contacts between each other in both low- and high-density monolayers, which makes it difficult to visualize the cell boundaries by phase-contrast microscopy (Fig. 4*B*). In contrast, IBMX-treated ECs failed to make such intimate contacts. The formation of spatial gaps was evident between these ECs, consistent with the weakened adherens junctions (Fig. 4*A*). Despite these changes, the total VE-cadherin protein level in ECs did not decrease on cAMP elevation (Fig. 4*C*). Western blot analysis detected total (clustered or nonclustered) VE-cadherin in the cell lysate. However, the immunofluorescence of VE-cadherin by the standard staining method is not sensitive enough to detect sparsely distributed or internalized VE-cadherin, and strong VE-cadherin staining is only visualized when clustered at a high density in the stable adherens junctions (2, 9). To examine whether cAMP elevation changes the cellular distribution of VE-cadherin, we monitored the trafficking of cell-surface VE-cadherin by a mild acid wash assay (2). In this study, redistribution of VE-cadherin was traced by incubating live ECs with a mAb (BV6) that recognizes the extracellular domain of VE-cadherin. After a mild acid wash to strip the cell surface–bound antibodies, the acid-resistant vesicular staining pattern represented the intracellular vesicles containing VE-cadherin molecules that are internalized/endocytosed (Fig. 4*D*). This analysis revealed markedly increased VE-cadherin internalization in forskolin or IBMX-treated ECs. The combined results demonstrate that the prolonged cAMP elevation destabilizes endothelial adherens junctions.

**Figure 5:**
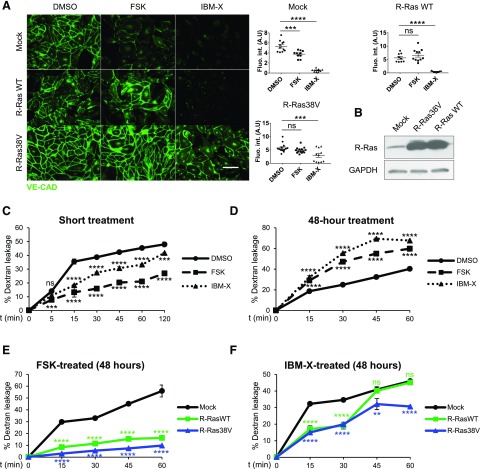
Forced expression of R-Ras restores endothelial barrier in ECs with elevated cAMP. *A*) Confluent monolayer of HUVECs transduced with mock, WT R-Ras (WT), or R-Ras38V were treated with DMSO, forskolin, or IBMX for 48 h and stained for VE-cadherin to assess junctional integrity. Integrated intensity of fluorescence signal was quantified in 3 distinct areas from at least 4 micrographs for each condition by ImageJ software. Scale bar, 100 µm. Ns, not significant. *P < 0.05, ***P < 0.001, ****P < 0.0001 (1-way ANOVA). *B*) R-Ras expression in HUVECs was analyzed by Western blot 4 d after transduction with mock, R-Ras38V, or R-Ras WT lentivirus. *C, D*) Endothelial permeability was assessed shortly after cAMP elevation (*C*) or in response to prolonged cAMP signaling (*D*). HUVECs were cultured in 24-well Transwell chambers (0.4 μm pore size) for 48 h to produce confluent monolayer. For short-term stimulation, cells were treated with DMSO, forskolin, or IBMX (*t* = 0 min), and FITC-dextran (70 kDa, 1 mg/ml) was added simultaneously into upper chamber. Fluorescence signal in lower compartment was measured by fluorophotometer at different time points (5–120 min after treatment). For long-term stimulation, permeability of HUVEC monolayer was determined after 48 h of cell incubation with DMSO, forskolin, or IBMX. FITC-dextran (70 kDa, 1 mg/ml) was subsequently added to upper chamber. Fluorescence signal in lower compartment was measured by fluorophotometer at different time points. Empty chambers without cells were used as reference (100% dextran leakage) for percentage calculation. *E, F*) Prolonged effects of cAMP signaling were analyzed using ECs transduced with mock, WT R-Ras, or R-Ras38V in forskolin- (*E*) or IBMX- (*F*) treated culture. Ns, not significant. **P < 0.01, ***P < 0.001, ****P < 0.0001 (2-way ANOVA).

Previously we showed that R-Ras promotes VE-cadherin clustering at adherens junctions *via* inhibition of VE-cadherin internalization without changing the total VE-cadherin protein level (2). The observed disruptions of VE-cadherin and EC-EC interaction by cAMP signaling are consistent with the observed down-regulation of R-Ras by the same signaling pathway (Fig. 1). Therefore, we examined whether the forced expression of R-Ras rescues the endothelial barrier stability from the disruption caused by cAMP elevation. HUVECs and HDMECs were transduced with WT or constitutively active R-Ras (R-Ras38V) and treated with forskolin or IBMX. The transduction of WT R-Ras restored the junctional VE-cadherin accumulation in the cells treated with forskolin, but not with IBMX (**Fig. 5*A***). The constitutively active R-Ras38V had a stronger effect, stabilizing VE-cadherin against the disruption by forskolin or by IBMX (Fig. 5*A*).

VEGF-A is a major angiogenic factor that signals *via* VEGFR2 activation and potently induces vascular permeability by disrupting VE-cadherin and destabilizing adherens junctions between adjacent ECs (9). We previously showed that R-Ras inhibits VEGF-A–induced VEGFR2 activation in ECs (4). In the current study, we observed increased expression of VEGF-A in ECs after 24 h of treatment with forskolin (Fig. 4*E*). This effect of forskolin has been reported previously (36). Notably, the concentration of VEGF-A^165^ was increased by 22-fold in the conditioned media of these ECs (Fig. 4*F*). These observations suggest an additional cAMP-responsive gene regulation critically contributing to the disruption of endothelial integrity and increased permeability synergizing with the *RRAS* repression effect of cAMP.

### cAMP signaling ultimately increases vascular permeability

To determine the role of cAMP in endothelial permeability, we first examined the short-term effect of cAMP elevation. The permeability of confluent HUVEC monolayer was measured by diffusion of FITC-dextran across the culture inserts. In this analysis, the EC permeability decreased promptly after the addition of forskolin or IBMX (Fig. 5*C* and Supplemental Fig. S2). This observation confirms that the short-term effect of cAMP elevation is endothelial barrier protective (18, 29, 30).

**Figure 6:**
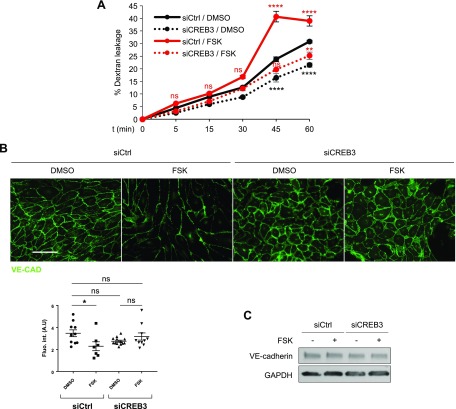
CREB3 knockdown restores endothelial barrier function. *A*) Permeability of control (siCtrl) or CREB3-silenced (siCREB3) HUVEC monolayer was determined using Transwell chamber system after 48 h of cell incubation with DMSO or forskolin. FITC-dextran (70 kDa, 1 mg/ml) was added to upper chamber, and fluorescence signal in lower compartment was measured by fluorophotometer at different time points. Empty chambers without cells were used as reference (100% dextran leakage) for percentage calculation. Ns, not significant.**P < 0.01, ****P < 0.0001 (3-way ANOVA). *B*) Confluent monolayer of control (siCtrl) or CREB3-silenced (siCREB3) HUVEC was treated with DMSO or forskolin for 48 h and subsequently stained for VE-cadherin. Quantification of immunostaining was performed by measuring integrated fluorescence intensity of 3 different areas from at least 4 micrographs for each condition. Scale bar, 100 µm. Ns, not significant. *P < 0.05 (2-way ANOVA). *C*) VE-cadherin expression in CREB3-silenced ECs in DMSO control or forskolin-treated culture was analyzed by Western blot.

We next investigated the long-term effect of cAMP elevation. Forskolin or IBMX was added to confluent monolayer of HUVECs or HDMECs in culture inserts, and FITC-dextran leakage was measured 48 h later. In this study, both forskolin and IBMX treatments increased EC permeability (Fig. 5*D* and Supplemental Fig. S3*A*). These observations are consistent with the VE-cadherin disruption and junctional instability of ECs observed 48 h after the treatments with these agents (Fig. 4). Because cAMP signaling transcriptionally down-regulates the expression of endogenous R-Ras, we examined whether a forced expression of R-Ras reverses the cAMP-caused EC permeability. The R-Ras transduction counteracted the barrier-disrupting effect of cAMP and reduced endothelial permeability (Fig. 5*E, F* and Supplemental Fig. S3*B, C*). CREB3 knockdown, which restores R-Ras expression in forskolin-treated cells (Fig. 4), had similar effects. Upon CREB3 knockdown, VE-cadherin was restored at the adherens junctions, and the permeability was reduced in the EC monolayer treated with forskolin for 48 h (**Fig. 6*A, B***). CREB3 knockdown had no effect on the expression of VE-cadherin (Fig. 6*C*), consistent with the role of R-Ras in VE-cadherin regulation (2).

**Figure 7:**
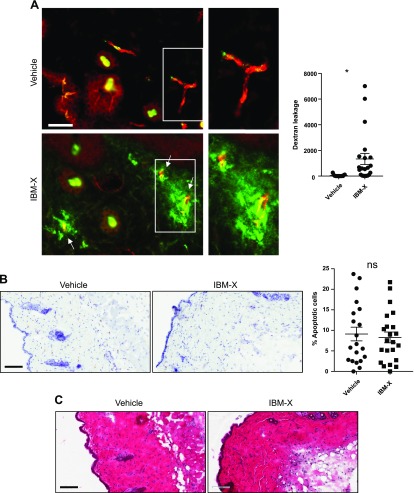
Prolonged cAMP elevation results in plasma leakage from dermal microvessels. *A*) Mice were injected with IBMX (300 µg/kg) or vehicle (DMSO) into dorsal skin on d 0 and d 1, then received intravenous injection of FITC-dextran (1 mg/ml) on d 2 to examine vessel permeability. Skin samples were collected 30 min after FITC-dextran injection, and tissue sections were immunostained with EC marker CD31 (red). Plasma leakage was assessed by quantitative fluorescence analysis of FITC-dextran outside of vessels (arrows). Hair follicles have green autofluorescence (asterisk). Degree of dextran leakage is presented in graph using arbitrary units. Analysis was conducted in 15 ∼ 21 micrographs of different areas in 4 skin samples for each condition. *P < 0.05 (Student’s *t* test). Scale bar, 40 µm. *B*) Toxicity of IBMX was evaluated in dorsal skin tissue sections by immunohistochemical staining of cleaved caspase-3 to detect apoptotic cells (brown). Percentage of apoptotic cells was determined by ratio between apoptotic and total cell numbers in micrographs of at least 20 different areas in 4 skin samples for each condition. ns, not significant. *C*) Histologic examination did not indicate accumulation of infiltrating immune cells in IBMX injected area. Scale bars, 100 µM.

Next, we investigated the long-term effect of cAMP on intact microvessels in mice. Mice received an intradermal injection of IBMX (300 µg/kg) in the dorsal skin. The injection was provided twice over 48 h, followed by intravenous injection of FITC-dextran from the tail vein. The cryosections of mouse skin were stained for CD31 and examined for vessel permeability by measuring FITC-dextran leakage around the vessels. Quantitative image analyses revealed a significant dextran leakage from the vessels in the skin injected with IBMX (**Fig. 7*A***). In comparison, essentially no leakage was detected in the control skin that received vehicle only (DMSO). To rule out the possibility that the observed results are due to IBMX toxicity, we analyzed cell apoptosis in the skin tissue sections by immunostaining of cleaved caspase-3. This analysis did not find increased cell apoptosis in the skin injected with IBMX (Fig. 7*B*). Furthermore, histologic evaluations of the skin revealed no sign of tissue abnormalities (Fig. 7*C* and Supplemental Fig. S4). These results indicate that the observed vascular permeability is not due to drug toxicity or tissue damage. Taken together, our results demonstrated that the long-term effect of cAMP elevation increases endothelial permeability.

### PGE2 down-regulates R-Ras and increases permeability of microvascular ECs

**Figure 8:**
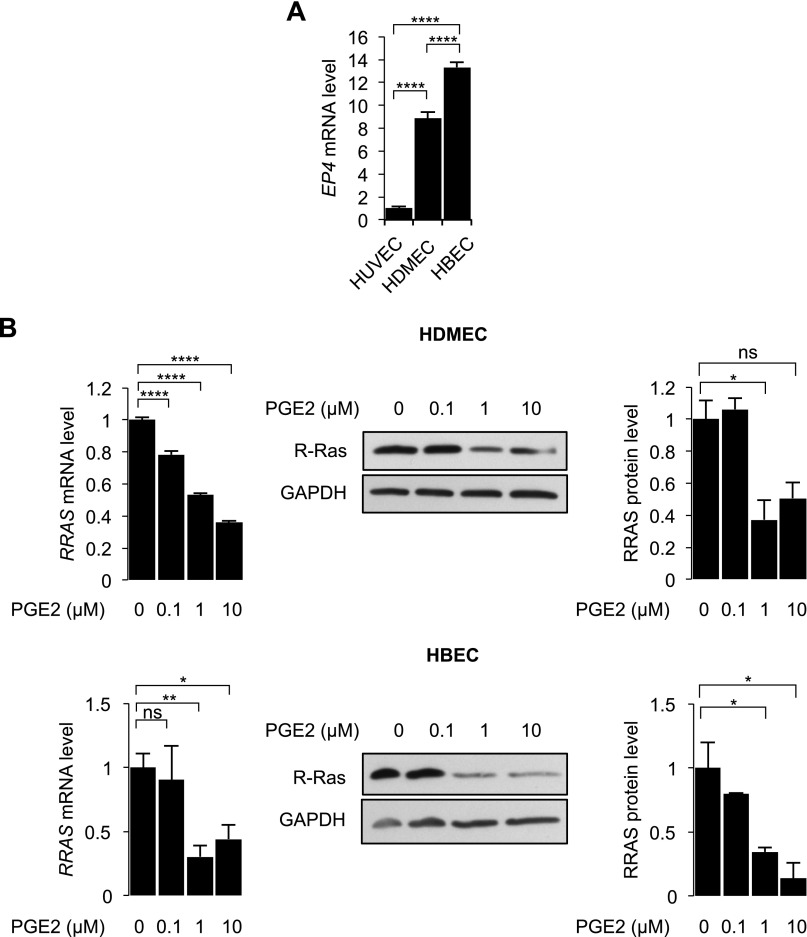
PGE2 down-regulates *RRAS* in EP_4_-expressing ECs. *A*) Level of EP_4_ expression was compared between HUVECs, HDMECs, and HBECs by qRT-PCR. *B*) HDMECs (top) and HBECs (bottom) were treated with increasing concentrations of PGE2 (0.1, 1, and 10 µM), and *RRAS* expression was analyzed by qRT-PCR at 48 h and by Western blot at 72 h after PGE2 treatment. Ns, not significant. ****P < 0.0001, **P < 0.01, *P < 0.05 (1-way ANOVA).

The intracellular cAMP level is elevated *via* activation of GPCRs (37, 38). Therefore, we investigated the effect of cAMP signaling induced by a natural ligand for GPCRs. PGE2 is a proinflammatory lipid that binds to and activates GPCRs, such as EP_4_, and increases cAMP production by activating AC (39, 40). PGE2 is known to increase vascular permeability (41–43) as well as angiogenesis (44). We first studied the EP_4_ expression levels in HUVECs, HDMECs, and HBECs to evaluate their responsiveness upon PGE2 stimulation. We observed that EP_4_ is expressed in both microvascular EC types, HDMECs and HBECs, but that EP_4_ expression in HUVECs is negligible (**Fig. 8*A***). These ECs were treated with increasing concentrations of PGE2, and R-Ras expression was assessed by qRT-PCR and Western blot analyses at 48 and 72 h after treatment, respectively. These experiments demonstrated that PGE2 down-regulates R-Ras in HDMECs and HBECs (Fig. 8*B*). In contrast, PGE2 did not repress R-Ras expression in HUVECs (data not shown). This observation is consistent with the very low expression of the PGE2 receptor EP_4_ in HUVECs compared to the other 2 EC types (Fig. 8*A*).

**Figure 9:**
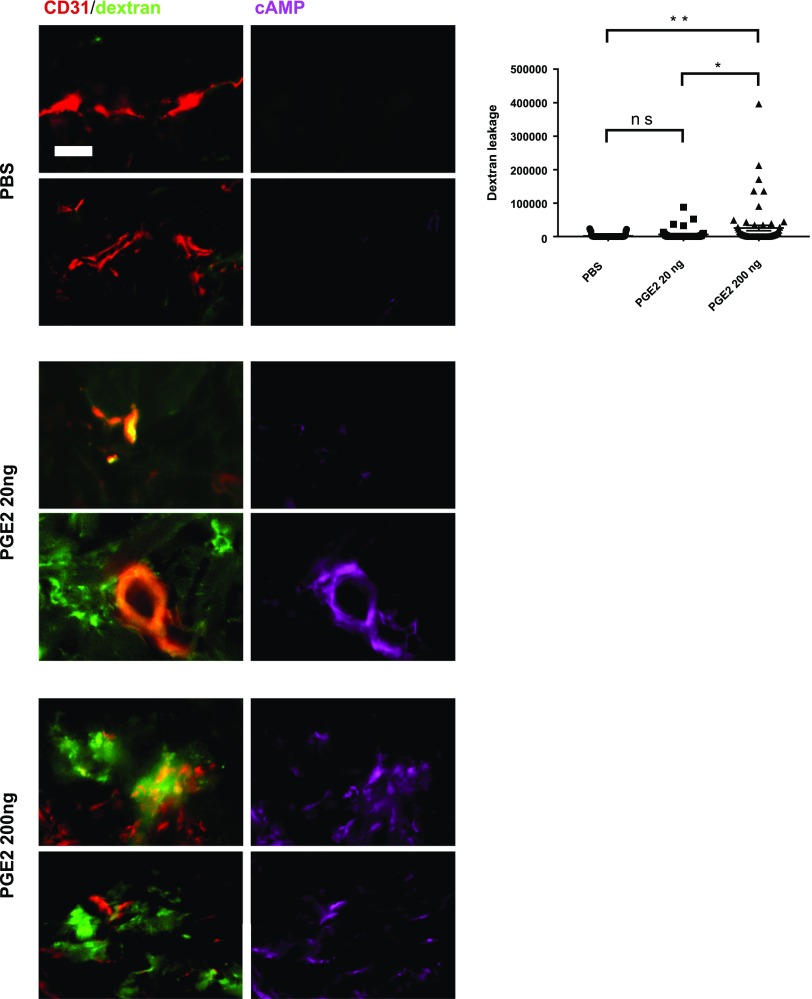
PGE2 elevates intracellular cAMP in ECs and induces permeability of dermal microvessels. Mice were injected with PGE2 (20 or 200 ng) or vehicle (DMSO) into dorsal skin on d 0 and d 1, then received intravenous injection of FITC-dextran (1 mg/ml) on d 2 to examine vessel permeability. Skin samples were collected 30 min after FITC-dextran injection, and tissue sections were immunostained for CD31 (red) and cAMP (magenta). Two representative micrographs of microvessels are shown for each condition and staining. Plasma leakage was assessed by quantitative fluorescence analysis of FITC-dextran outside of vessels. Degree of dextran leakage is presented in graph using arbitrary units. Analysis was conducted in >40 micrographs of different areas in 4 skin samples for each condition. Scale bar, 20 µm. Ns, not significant. *P < 0.05, **P < 0.01 (1-way ANOVA).

Next, we examined vascular leakage in dermal microvessels in response to a PGE2 exposure *in vivo*. Mice received PGE2 (20 or 200 ng) intradermally in the dorsal skin, and the injection was repeated once in the same area 24 h later. At 48 h after the first injection, mice received an intravenous injection of FITC-dextran at the tail vein. Mouse skin sections were stained for CD31 and cAMP, and examined for vascular permeability by quantifying the FITC-dextran leakage around vessels. Consistent with the report of Omori *et al.* (41), PGE2 administration at 200 ng resulted in significant dextran leakage from some microvessels (**Fig. 9**). Dextran leakage was also observed occasionally after the treatment with a lower dose (20 ng) of PGE2, albeit a statistically insignificant amount of leakage. The PGE2-induced dextran leakage was clearly associated with the cAMP elevation in the endothelium of microvessels (Fig. 9). Thus, all cAMP-elevated vessels exhibited dextran leakage. These results strongly support that the long-term cAMP elevation in ECs due to a chronic exposure to PGE2 leads to hyperpermeability of the microvasculature.

### Constant CREB activation and R-Ras down-regulation in the tumor vasculature

**Figure 10:**
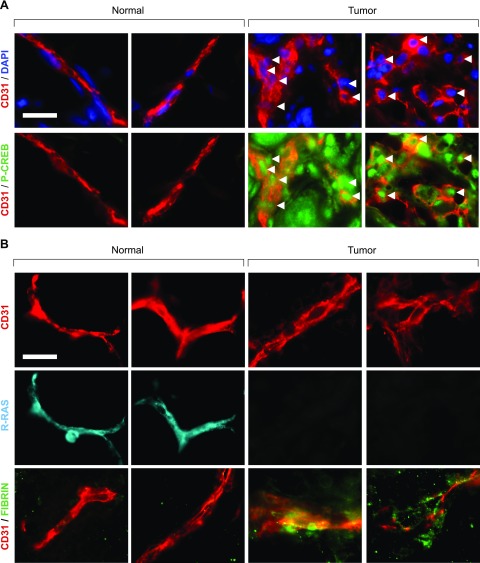
Tumor vessels display cAMP pathway activation, R-Ras down-regulation, and hyperpermeability in human breast cancer. *A*) Normal (left) and tumor (right) breast tissue sections were stained for DAPI (blue), CD31 (red), and phospho-CREB1 (green) as indicator of cAMP signaling activation. In tumor sections, intense phospho-CREB1 staining was observed in EC nuclei (white arrows). *B*) Same sections were stained for CD31 (red), R-Ras (cyan), and fibrin (green) to assess plasma leakage. Tumor vessels exhibited loss of R-Ras expression and presence of plasma leakage. Breast cancer samples from 4 different patients were examined, and 2 representative micrographs are shown for each staining. Scale bar, 20 µm.

Increased permeability is a typical characteristic of tumor vasculature. PGE2 is the most abundant prostaglandin found in the tumor microenvironment in various malignancies, and it is associated with poor prognosis in breast cancer and other cancer types (45). PGE2 released from mammary tumor cells was shown to promote angiogenesis through the induction of proangiogenic factors *via* a cAMP-PKA-dependent pathway *in vitro* and *in vivo* (46). We investigated the activation of the cAMP signaling in tumor vascular ECs in human breast cancer tissues from patients. The sections of breast cancer tissues and noncancerous normal portion of the breast tissues from the same patients were stained for CD31 and phospho-CREB1 (serine 133). Because cAMP signaling leads to CREB1 phosphorylation that can be easily determined *in situ* by immunostaining, we used this method to assess the activation of cAMP signaling. We observed that both cancer cells and tumor vascular ECs display intense nuclear staining of phospho-CREB1, indicating chronic activation of the cAMP-PKA cascade in these cells (**Fig. 10*A***). Consistent with our previous report (2), we also observed the absence of R-Ras expression in tumor blood vessels and concomitant blood leakage, as indicated by fibrin accumulation around the vessels (Fig. 10*B*). These results demonstrate that the cAMP signaling is chronically activated in the tumor vasculature, and this activation is associated with R-Ras down-regulation and increased vascular permeability.

## DISCUSSION

R-Ras plays a crucial role in the stabilization of blood vessel wall during vascular regeneration (2). A strong down-regulation of R-Ras is associated with leaky blood vessels in human malignant tumors and diabetic retinopathy (2, 10). In this study, we demonstrated that the activation of cAMP signaling results in CREB3-dependent down-regulation of *RRAS* in both macrovascular and microvascular ECs. We identified 3 CREs within the *RRAS* promoter sequence that are necessary for the cAMP-responsive transcriptional repression. An RNAi analysis revealed that CREB3 is the most important among 3 CREB proteins for mediating the inhibitory effect of cAMP on *RRAS* transcription. Moreover, our RNA sequencing analysis revealed that CREB3 is expressed >10-fold more abundantly than CREB1 or ATF2 in ECs, further supporting the importance of CREB3 for EC regulation. Consistent with the down-regulation of *RRAS* mRNA and R-Ras protein, the activation of cAMP signaling caused the loss of VE-cadherin accumulation at adherens junctions and the disruption of EC-EC interaction, resulting in a marked increase of endothelial permeability. These effects of cAMP were offset by rescuing R-Ras expression by cDNA transduction or CREB3 silencing. In agreement with these *in vitro* observations, we observed that elevation of cAMP by intradermal administration of IBMX in mice resulted in considerable plasma leakage from dermal microvessels. To our knowledge, this is the first study reporting the cAMP-dependent endothelial barrier disruption *in vivo*. PGE2 is a physiologic activator of cAMP signaling and a major prostanoid abundantly produced on acute or chronic inflammation. We showed that PGE2 suppresses R-Ras expression in ECs and induces vascular permeability. Our results suggest a role for PGE2 in abnormal vascular permeability caused by the long-term activation of cAMP signaling in pathologic conditions.

Our study demonstrated that the overall long-term consequence of cAMP elevation in ECs is a significant increase of endothelial permeability. This finding was unexpected because it has been widely accepted that cAMP is endothelial barrier protective and that the elevation of intracellular cAMP level reduces endothelial permeability (18, 21, 47). However, it is important to note that the barrier-protective effect of cAMP in previous studies was demonstrated in a short time after cAMP elevation. Thus, these observations represent an immediate response of ECs to the cAMP signaling promptly modulating actin cytoskeleton, cell contraction, and tight/adherens junctional complexes (18, 19, 29, 30). Interestingly, cAMP increases R-Ras activity in ECs (48), potentially contributing to the early barrier-protection response. However, we observed that this activation is a transient effect that lasts no longer than 2 h (data not shown). These observations suggest that the rapid modulation of EC permeability by cAMP is barrier protective. On the other hand, delayed responses to cAMP elevation are mediated *via* cAMP-dependent gene regulations. Upon induction of cAMP signaling, CREB3 represses *RRAS* gene expression, leading to weakened VE-cadherin clustering and increased EC permeability. We also showed that cAMP elevation increases the production of VEGF-A by ECs, as previously reported (36). Because VEGF-A is a potent vascular permeability factor (49), this observation provides an additional mechanism of endothelial barrier disruption by cAMP-dependent gene expression. R-Ras antagonizes the VEGF-A signaling in ECs by suppressing VEGFR2 activation (4). The current study revealed that cAMP represses R-Ras and enhances VEGF-A expression simultaneously, suggesting a synergy of the 2 effects of cAMP signaling on vascular permeability.

Another important factor to determine the 2 opposing (barrier protective *vs.* disruptive) effects of cAMP may be the subcellular location of cAMP accumulation in ECs. It is postulated that the accumulation of cAMP is highly compartmentalized in different pools within the cell (50, 51). Growing evidence suggests that cAMP accumulation in the membrane compartment and in the cytosol can have barrier-stabilizing and -disrupting effects, respectively (23, 24). PDEs appear to compartmentalize cAMP and regulate a membrane-to-cytosol cAMP gradient by hydrolyzing cAMP before it disseminates from the membrane periphery to the cytosol (52). Because the effectors for cAMP-dependent gene regulations (*e.g.*, PKA and CREB) are present in cytosol, this cAMP gradient model could explain why the PDE inhibitor IBMX, which allows overflowing of cAMP into cytosol, was more effective than AC activator forskolin in disrupting endothelial barrier in our study.

Our new finding is particularly important for its relevance in pathologic conditions associated with abnormally increased extracellular stimuli for cAMP signaling. For instance, prostaglandins are highly elevated in the tumor stroma of many types of cancer, including breast cancer (45, 53–55). We found nuclear accumulation of phosphorylated CREB1 in the tumor vascular endothelium of human breast cancer, indicating chronic activation of cAMP signaling in these vessels. Therefore, the high PGE2 levels in tumors could explain the down-regulation of R-Ras and associated leakiness of the tumor vasculature (2). Other cAMP-activating factors that are found abnormally increased in tumor stroma are extracellular adenosine (56) and stress hormones such as norepinephrine (57–60). These factors could chronically elevate cAMP signaling in the tumor vasculature. The increased vascular permeability facilitates tumor metastasis (5, 61) and raises interstitial fluid pressure to hinder deep penetration of cancer drugs in tumors (5, 62). The elevation of cAMP signaling in tumor vascular ECs has an important implication in cancer progression and therapy.

**Figure 11:**
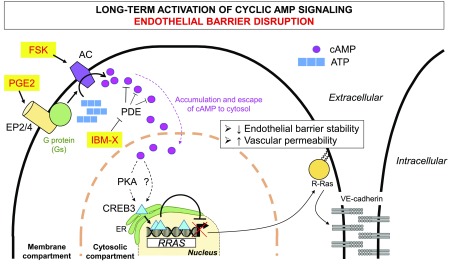
Working model for endothelial barrier instability by cAMP-dependent *RRAS* gene repression. Activation of PGE2 receptors, AC activation by forskolin, or PDE inhibition results in accumulation of cAMP at membrane periphery (membrane compartment) followed by diffusion of cAMP into cytosolic compartment. This process leads to release and activation of endoplasmic reticulum–bound transcription factor CREB3 through PKA-dependent and possibly PKA-independent mechanisms. Activated CREB3 then translocates to nucleus, where it binds to 3 CREs within *RRAS* promoter to inhibit *RRAS* expression. Down-regulation of R-Ras destabilizes VE-cadherin–dependent adherens junction formation, resulting in increased vascular permeability.

In summary, our study revealed that the long-term effect of cAMP signaling is detrimental to the endothelial barrier integrity and causes pronounced vessel leakiness. This effect is mediated *via* cAMP-responsive regulation of *RRAS* and possibly other genes that are crucial to the endothelial permeability regulation (**Fig. 11**). Our findings suggest that the upstream regulators of cAMP signaling, such as ACs, GPCRs (*e.g.*, EP_4_ and β-adrenergic receptor), or enzymes or intermediates required for PGE2 synthesis, may be therapeutic targets for the intervention of abnormal vascular permeability. There are a number of existing small molecules to target these proteins, which are being or have been tested to treat various conditions. Some of these compounds may be repurposed for the use for controlling vessel leakiness.

## Supplementary Material

This article includes supplemental data. Please visit *http://www.fasebj.org* to obtain this information.

## Abbreviations

AC: adenylyl cyclase
ATF: activating transcription factor
ChIP: chromatin immunoprecipitation
CRE: cAMP response element
CREB: cAMP response element–binding
EBM-2: endothelial cell growth basal medium 2
EC: endothelial cell
EP_4_: prostaglandin E_2_ receptor 4
GTPase: guanosine triphosphate hydrolase
HBEC: human brain microvascular endothelial cell
HDMEC: human dermal microvascular endothelial cell
IBMX: 3-isobutyl-1-methylxanthine
MTT: (4,5-dimethylthiazol-2-*yl*)-2,5-diphenyltetrazolium bromide
PDE: phosphodiesterase
PGE2: prostaglandin E_2_
PKACα: catalytic subunit of human PKA
qPCR: quantitative PCR
R-Ras: Ras-related protein
siRNA: small interfering RNA
VEGFR2: VEGF receptor 2
WT: wild type
