# Supplementary material for: Prolonged activation of cAMP signaling leads to endothelial barrier disruption via transcriptional repression of RRAS
Source: FASEB J. 2018 May 18;32(11):5793–812. doi: 10.1096/fj.201700818RRR (PMC6181640; doi:10.1096/fj.201700818RRR)
Supplement: Supplementary file 1 [file fj.201700818RRR.sf1.pptx]

## Slide 1
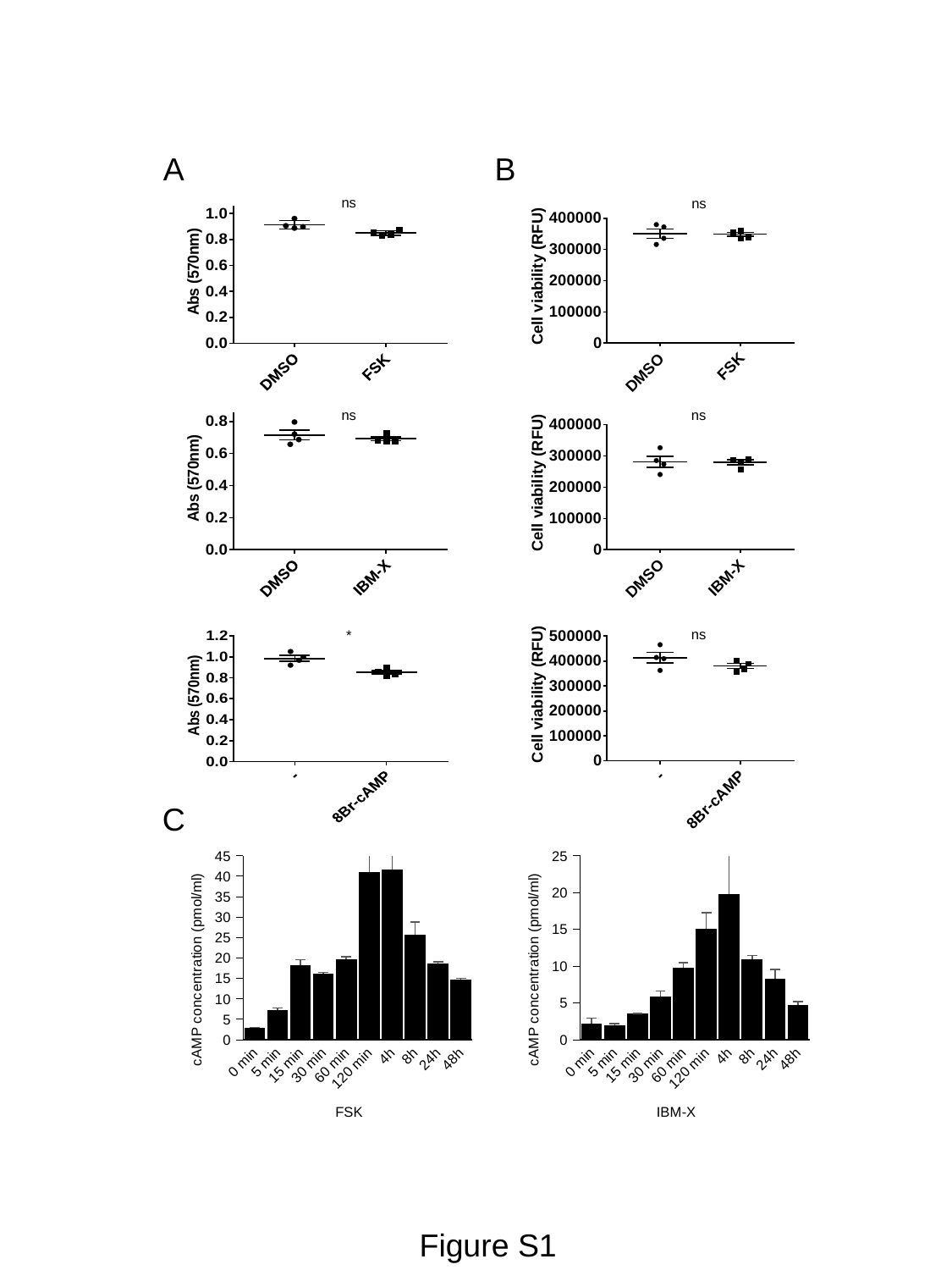

A
B
ns
ns
ns
ns
ns
*
C
### Chart
| Category | |
|---|---|
| 0 min | 2.934610639771531 |
| 5 min | 7.165063453334109 |
| 15 min | 18.1697892618113 |
| 30 min | 16.126109171749018 |
| 60 min | 19.789690825863513 |
| 120 min | 41.02911599455148 |
| 4h | 41.656916432801815 |
| 8h | 25.62780574004854 |
| 24h | 18.758987323533194 |
| 48h | 14.617117521459313 |
### Chart
| Category | |
|---|---|
| 0 min | 2.1867342099757443 |
| 5 min | 2.018299645709981 |
| 15 min | 3.519588599889328 |
| 30 min | 5.901575804373927 |
| 60 min | 9.76837765877613 |
| 120 min | 15.112612342366413 |
| 4h | 19.836250521025896 |
| 8h | 10.916653003120182 |
| 24h | 8.32179183827742 |
| 48h | 4.72141065044709 |FSK
IBM-X
Figure S1

## Slide 2
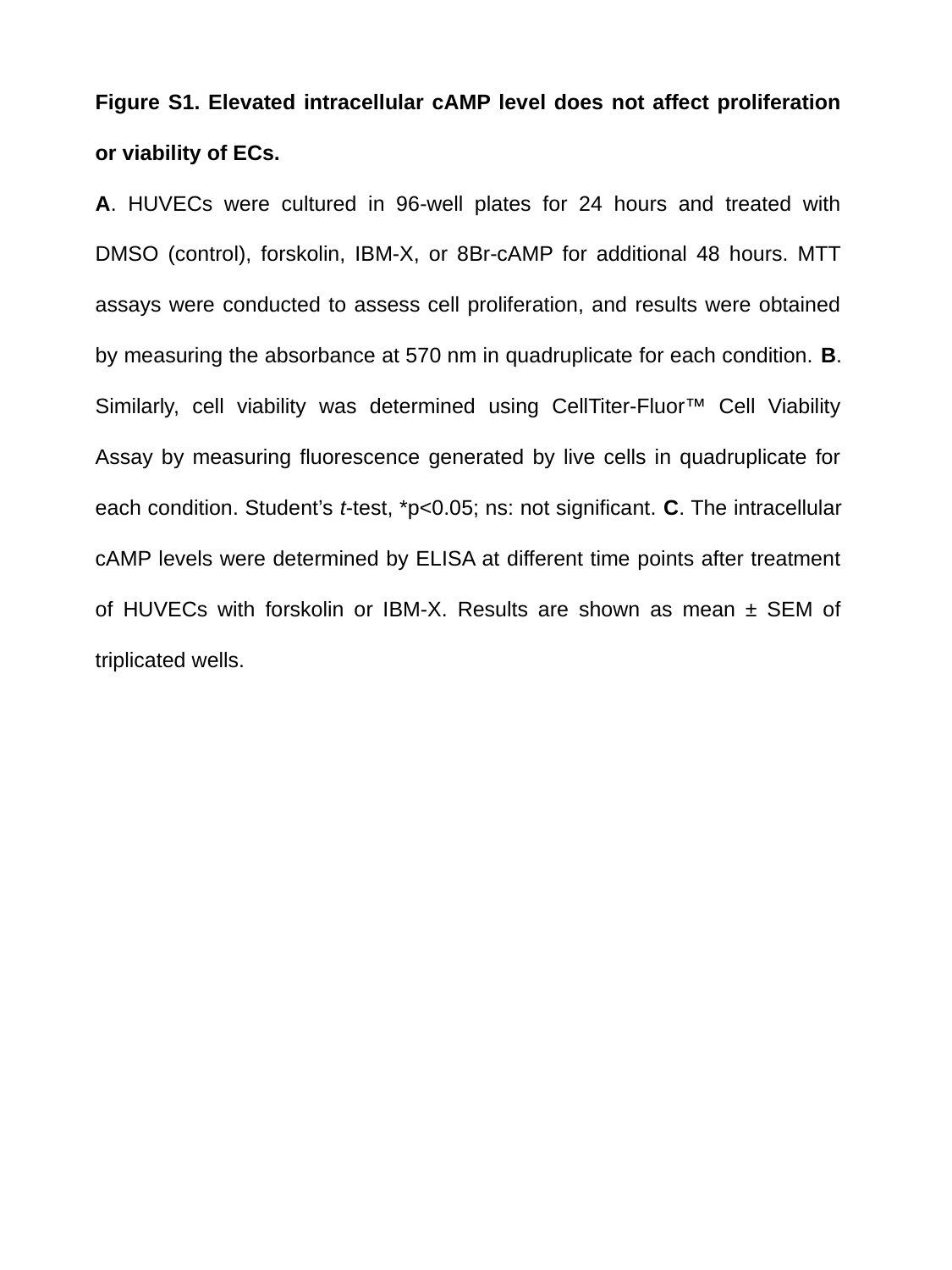

Figure S1. Elevated intracellular cAMP level does not affect proliferation or viability of ECs.
A. HUVECs were cultured in 96-well plates for 24 hours and treated with DMSO (control), forskolin, IBM-X, or 8Br-cAMP for additional 48 hours. MTT assays were conducted to assess cell proliferation, and results were obtained by measuring the absorbance at 570 nm in quadruplicate for each condition. B. Similarly, cell viability was determined using CellTiter-Fluor™ Cell Viability Assay by measuring fluorescence generated by live cells in quadruplicate for each condition. Student’s t-test, *p<0.05; ns: not significant. C. The intracellular cAMP levels were determined by ELISA at different time points after treatment of HUVECs with forskolin or IBM-X. Results are shown as mean ± SEM of triplicated wells.
