# Supplementary material for: Prolonged activation of cAMP signaling leads to endothelial barrier disruption via transcriptional repression of RRAS
Source: FASEB J. 2018 May 18;32(11):5793–812. doi: 10.1096/fj.201700818RRR (PMC6181640; doi:10.1096/fj.201700818RRR)
Supplement: Supplementary file 2 [file fj.201700818RRR.sf2.pptx]

## Slide 1
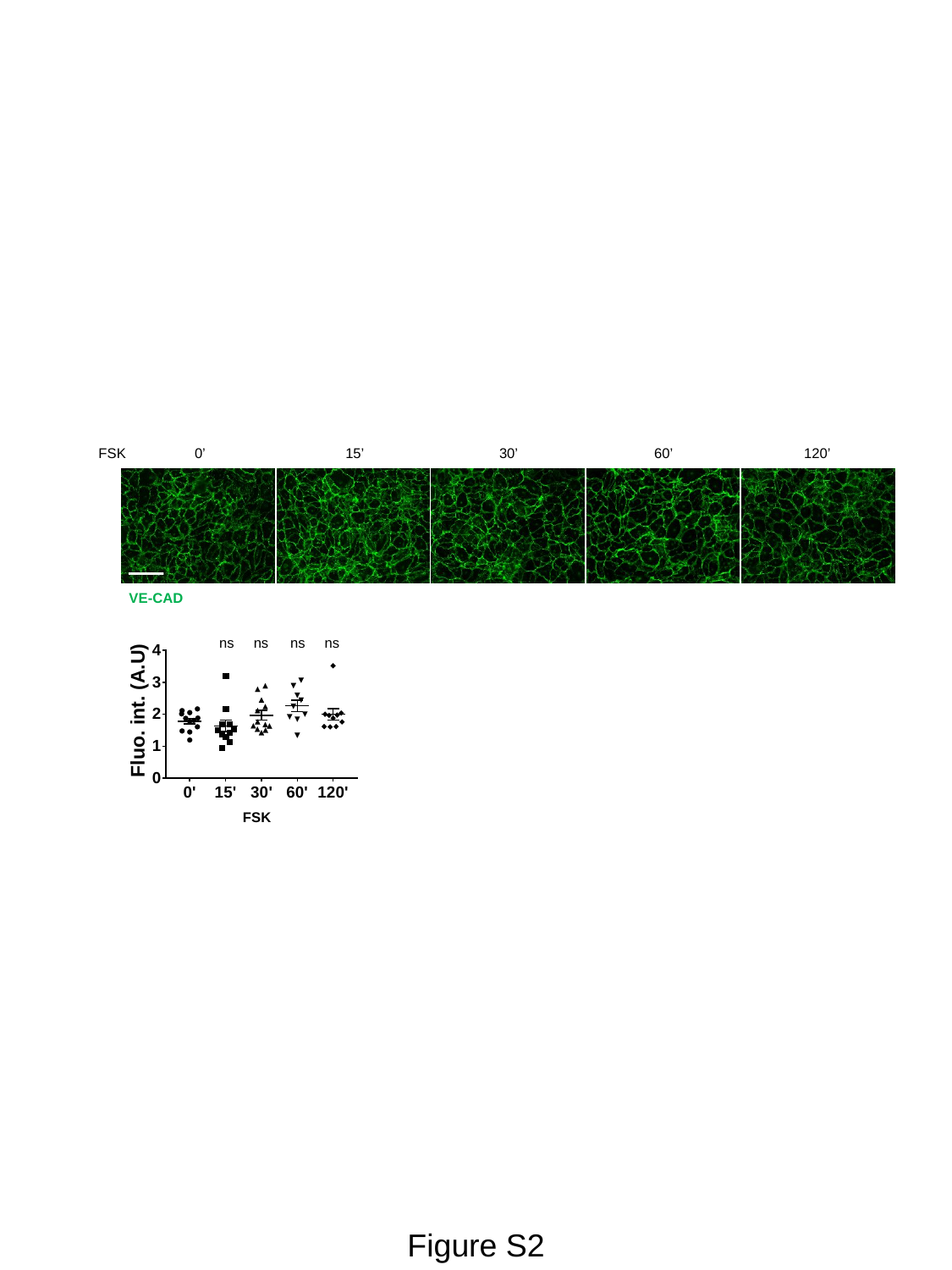

FSK
0’
15’
30’
60’
120’
VE-CAD
ns
ns
ns
ns
FSK
Figure S2

## Slide 2
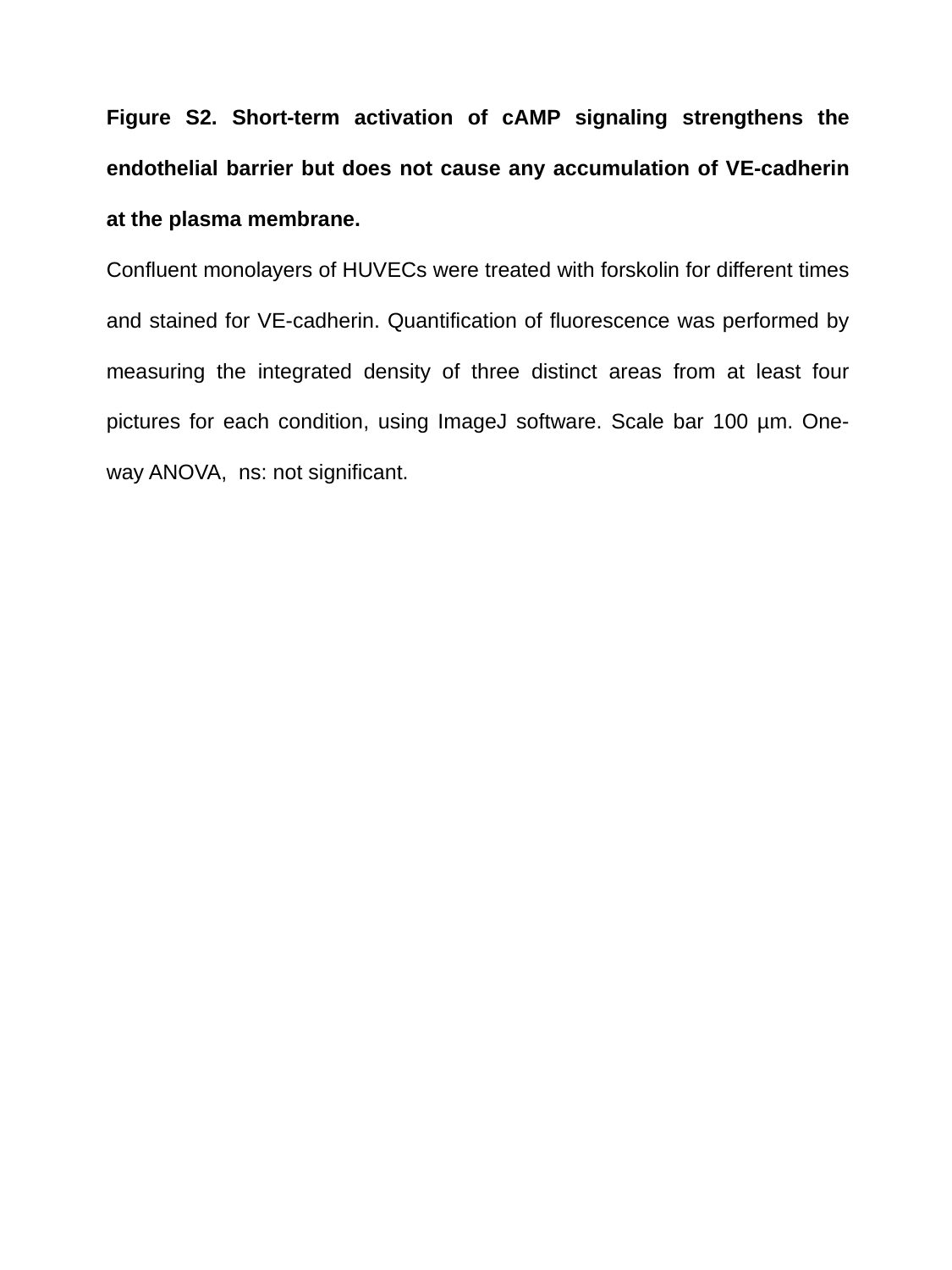

Figure S2. Short-term activation of cAMP signaling strengthens the endothelial barrier but does not cause any accumulation of VE-cadherin at the plasma membrane.
Confluent monolayers of HUVECs were treated with forskolin for different times and stained for VE-cadherin. Quantification of fluorescence was performed by measuring the integrated density of three distinct areas from at least four pictures for each condition, using ImageJ software. Scale bar 100 µm. One-way ANOVA, ns: not significant.
