# Supplementary material for: Prolonged activation of cAMP signaling leads to endothelial barrier disruption via transcriptional repression of RRAS
Source: FASEB J. 2018 May 18;32(11):5793–812. doi: 10.1096/fj.201700818RRR (PMC6181640; doi:10.1096/fj.201700818RRR)
Supplement: Supplementary file 3 [file fj.201700818RRR.sf3.pptx]

## Slide 1
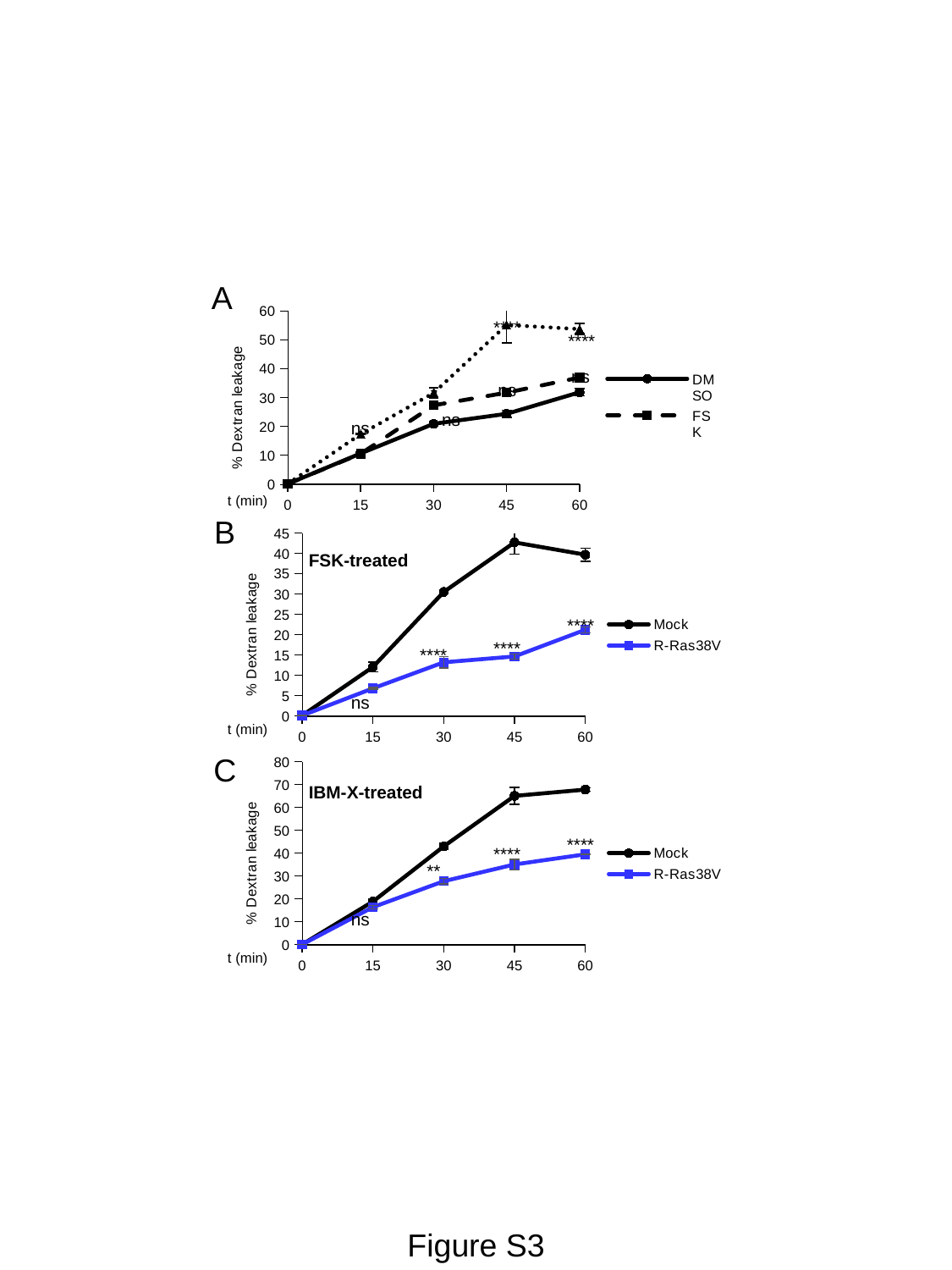

A
### Chart
| Category | | | |
|---|---|---|---|
| 0 | 0.14980410232772529 | 0.1555657985710993 | 0.16708919105784742 |
| 15 | 10.710993316432358 | 10.589997695321504 | 17.388799262502882 |
| 30 | 20.949527540908043 | 27.36229545978336 | 31.637474072366903 |
| 45 | 24.395021894445726 | 31.752707997234385 | 55.179764922793275 |
| 60 | 31.87370361834524 | 36.97280479373127 | 53.745102558193125 |****
****
ns
ns
*
ns
ns
t (min)
B
### Chart
| Category | | |
|---|---|---|
| 0 | 0.16132749481447337 | 0.17285088730122147 |
| 15 | 12.111085503572252 | 6.8564185296151186 |
| 30 | 30.571560267342704 | 13.251901359760314 |
| 45 | 42.734501037105325 | 14.68656372436045 |
| 60 | 39.715372205577324 | 21.254897441806868 |FSK-treated
****
****
****
ns
t (min)
C
### Chart
| Category | | |
|---|---|---|
| 0 | 0.16708919105784742 | 0.16708919105784742 |
| 15 | 18.927172159483753 | 16.472689559806405 |
| 30 | 43.062917722977645 | 27.81746946300991 |
| 45 | 65.11292924637013 | 35.1233002996082 |
| 60 | 67.86125835445955 | 39.58285319197972 |IBM-X-treated
****
****
**
ns
t (min)
Figure S3

## Slide 2
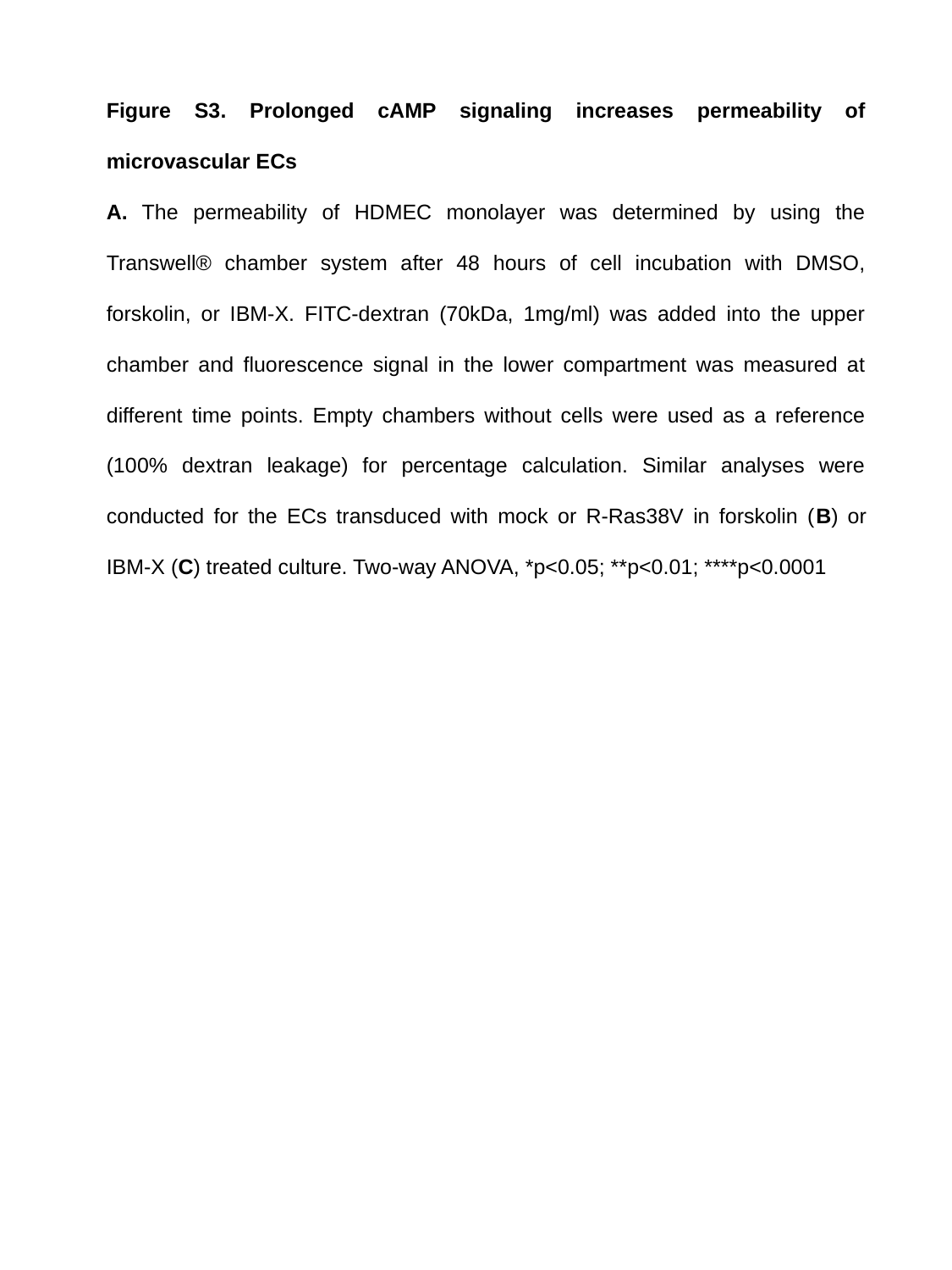

Figure S3. Prolonged cAMP signaling increases permeability of microvascular ECs
A. The permeability of HDMEC monolayer was determined by using the Transwell® chamber system after 48 hours of cell incubation with DMSO, forskolin, or IBM-X. FITC-dextran (70kDa, 1mg/ml) was added into the upper chamber and fluorescence signal in the lower compartment was measured at different time points. Empty chambers without cells were used as a reference (100% dextran leakage) for percentage calculation. Similar analyses were conducted for the ECs transduced with mock or R-Ras38V in forskolin (B) or IBM-X (C) treated culture. Two-way ANOVA, *p<0.05; **p<0.01; ****p<0.0001
