# Supplementary material for: Prolonged activation of cAMP signaling leads to endothelial barrier disruption via transcriptional repression of RRAS
Source: FASEB J. 2018 May 18;32(11):5793–812. doi: 10.1096/fj.201700818RRR (PMC6181640; doi:10.1096/fj.201700818RRR)
Supplement: Supplementary file 4 [file fj.201700818RRR.sf4.pptx]

## Slide 1
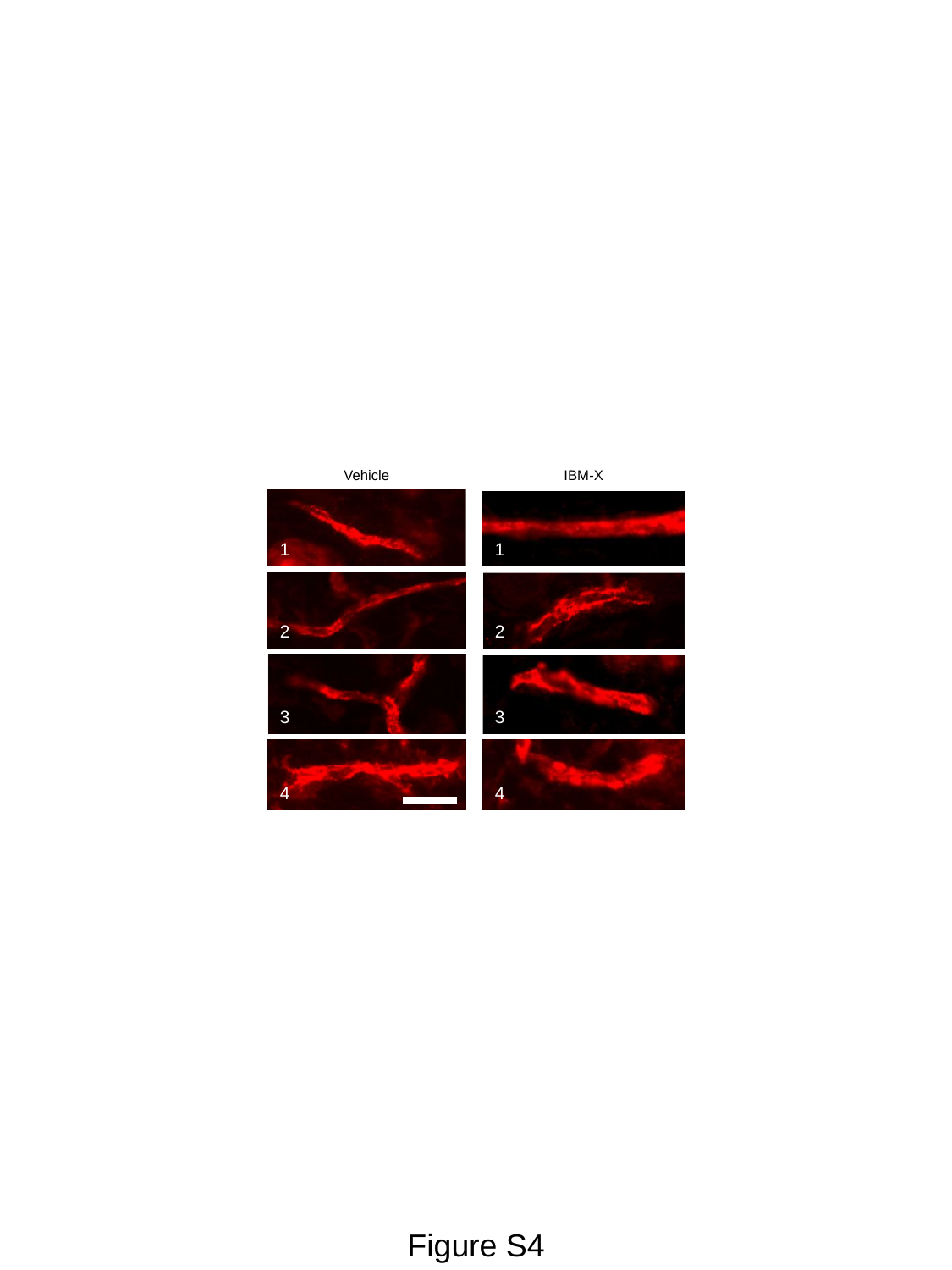

Vehicle
IBM-X
1
1
2
2
3
3
4
4
Figure S4

## Slide 2
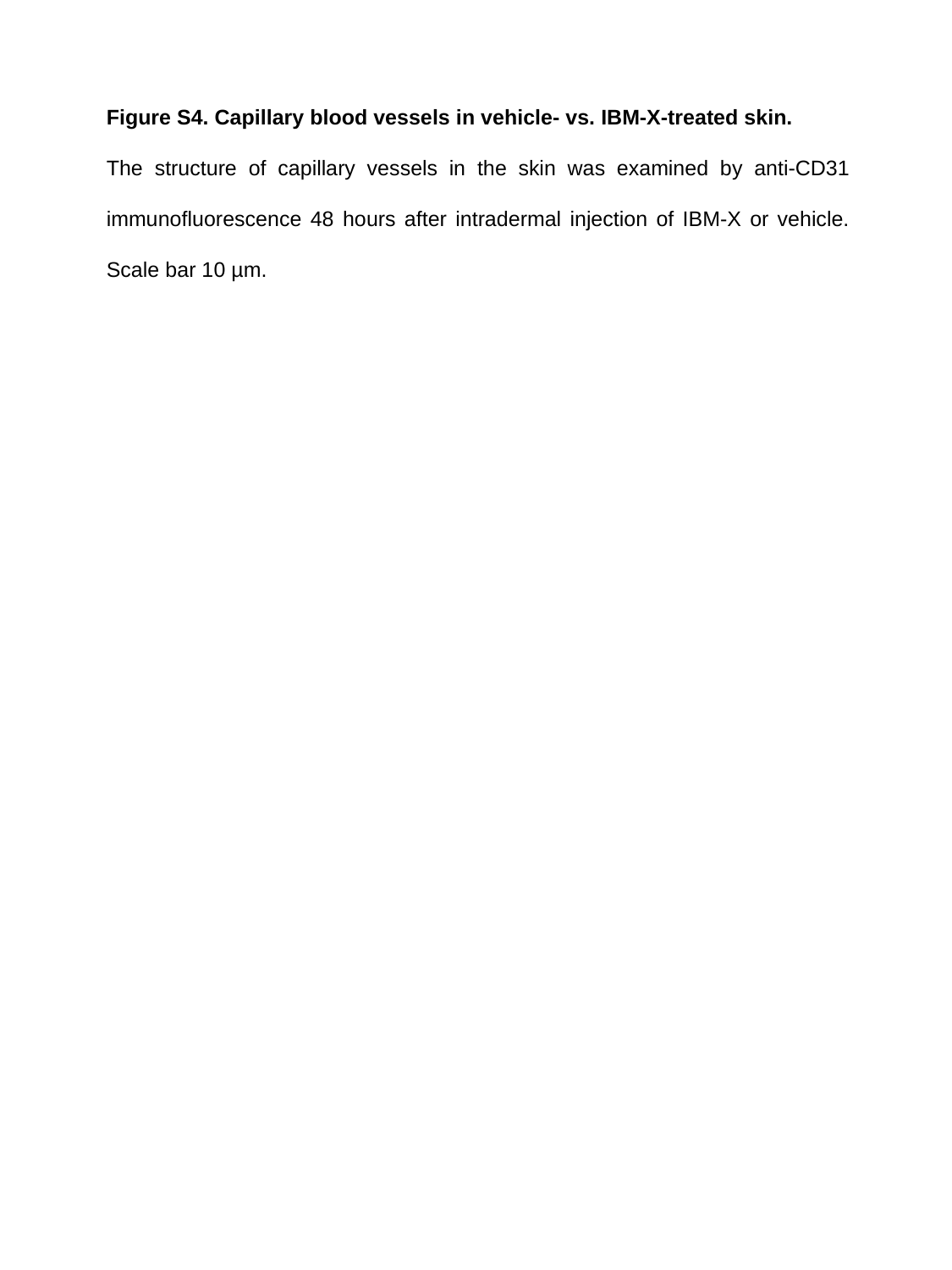

Figure S4. Capillary blood vessels in vehicle- vs. IBM-X-treated skin.
The structure of capillary vessels in the skin was examined by anti-CD31 immunofluorescence 48 hours after intradermal injection of IBM-X or vehicle. Scale bar 10 µm.
